# Host Reticulocytes Provide Metabolic Reservoirs That Can Be Exploited by Malaria Parasites

**DOI:** 10.1371/journal.ppat.1004882

**Published:** 2015-06-04

**Authors:** Anubhav Srivastava, Darren J. Creek, Krystal J. Evans, David De Souza, Louis Schofield, Sylke Müller, Michael P. Barrett, Malcolm J. McConville, Andrew P. Waters

**Affiliations:** 1 Wellcome Trust Centre for Molecular Parasitology, College of Medical, Veterinary and Life Sciences, University of Glasgow, Scotland, United Kingdom; 2 Institute of Infection, Immunity & Inflammation, College of Medical, Veterinary and Life Sciences, University of Glasgow, Scotland, United Kingdom; 3 Drug Delivery, Disposition and Dynamics, Monash Institute of Pharmaceutical Sciences, Monash University, Parkville, Australia; 4 Walter and Eliza Hall Institute of Medical Research, Division of Infection and Immunity, Parkville, Victoria, Australia; 5 Metabolomics Australia, Bio21 Molecular Science and Biotechnology Institute, University of Melbourne, Parkville, Victoria, Australia; 6 Australian Institute of Tropical Health and Medicine, Centre for Biodiscovery and Molecular Development of Therapeutics, James Cook University, Townsville, Australia; 7 Department of Biochemistry and Molecular Biology, University of Melbourne, Parkville, Victoria, Australia; Case Western Reserve University, UNITED STATES

## Abstract

Human malaria parasites proliferate in different erythroid cell types during infection. Whilst *Plasmodium vivax* exhibits a strong preference for immature reticulocytes, the more pathogenic *P*. *falciparum* primarily infects mature erythrocytes. In order to assess if these two cell types offer different growth conditions and relate them to parasite preference, we compared the metabolomes of human and rodent reticulocytes with those of their mature erythrocyte counterparts. Reticulocytes were found to have a more complex, enriched metabolic profile than mature erythrocytes and a higher level of metabolic overlap between reticulocyte resident parasite stages and their host cell. This redundancy was assessed by generating a panel of mutants of the rodent malaria parasite *P*. *berghei* with defects in intermediary carbon metabolism (ICM) and pyrimidine biosynthesis known to be important for *P*. *falciparum* growth and survival *in vitro* in mature erythrocytes. *P*. *berghei* ICM mutants (*pbpepc^-^*, phosphoenolpyruvate carboxylase and *pbmdh^-^*, malate dehydrogenase) multiplied in reticulocytes and committed to sexual development like wild type parasites. However, *P*. *berghei* pyrimidine biosynthesis mutants (*pboprt^-^*, orotate phosphoribosyltransferase and *pbompdc^-^*, orotidine 5′-monophosphate decarboxylase) were restricted to growth in the youngest forms of reticulocytes and had a severe slow growth phenotype in part resulting from reduced merozoite production. The *pbpepc^-^*, *pboprt^-^* and *pbompdc^-^* mutants retained virulence in mice implying that malaria parasites can partially salvage pyrimidines but failed to complete differentiation to various stages in mosquitoes. These findings suggest that species-specific differences in *Plasmodium* host cell tropism result in marked differences in the necessity for parasite intrinsic metabolism. These data have implications for drug design when targeting mature erythrocyte or reticulocyte resident parasites.

## Author Summary

Malaria, caused by the Apicomplexan parasites *Plasmodium* spp., is a deadly disease which poses a huge health and economic burden over many populations in the world, mostly in sub-Saharan Africa and Asia. To design new intervention strategies and to improve upon existing drugs against malaria, it is useful to understand the biochemistry of the *Plasmodium* parasite and its metabolic interplay with the host. Some species of *Plasmodium* such as *P*. *vivax* grow exclusively in reticulocytes (immature erythrocytes) whereas others e.g. *P*. *falciparum* will also readily multiply in mature erythrocytes. We asked the questions, do these two classes of host cell offer different resources for parasite survival and could these resources influence antimalarial drug efficacy? We used metabolomics to compare rodent reticulocytes and mature erythrocytes and identified that the metabolome of the former is more diverse and enriched. Gene disruption in the reticulocyte preferring rodent malaria parasite *P*. *berghei* was used to demonstrate that *Plasmodium* can utilise the elements of the metabolic reserves of reticulocytes that mature erythrocytes cannot provide. Our data suggests that the availability of the reticulocyte metabolome might reduce or block the efficacy of antimalarial drugs that target parasite metabolism and drugs tested against *P*. *falciparum* might have significantly reduced activity against *P*. *vivax*.

## Introduction

The malaria-causing apicomplexan parasites *Plasmodium* spp. have a dynamic life cycle which is reflected in stage-specific morphologies, transcriptomes, proteomes and metabolomes [1–8]. These changes, particularly in their metabolome, reflect the nutritional needs and biological processes of the parasite during intracellular development that in turn influences, or is influenced by, the physiological state of the host cell [6]. Perhaps due to their parasitic life-style, *Plasmodium* spp. have a simplified and reduced metabolic capacity when compared to higher non-parasitic organisms. They are auxotrophic for purines, vitamins and many amino acids [9,10], but have retained core pathways of carbon metabolism such as glycolysis [11], the citric acid cycle [7,12], lipid synthesis [13,14], the pentose phosphate pathway [15], pyrimidine biosynthesis [16] and glycosylation [17]. *Plasmodium* spp. are obligate intracellular parasites and their metabolism is interlinked with that of their host cell and is heavily dependent on the availability of external nutrients. As a result, intracellular *Plasmodium* establish systems such as the new permeation pathways with the purpose of accessing host cell and environmental nutrients [18]; in fact the parasite genome encodes >120 predicted membrane transport proteins, a subset of which are located on the plasma membrane [19].

Erythrocyte invasion is a prerequisite for establishment of infection by *Plasmodium* merozoites and the roles of different merozoite and host surface proteins in this invasion process have been intensively studied [20–25]. Multiple partially overlapping erythrocyte invasion pathways have been described in *P*. *falciparum* with consequent functional redundancy [26]. Many *Plasmodium* spp. including *P*. *falciparum* preferentially invade reticulocytes [27] which is also capable of invading and replicating within all stages of erythrocyte development including mature cells. However, *P*. *vivax* has a strict requirement for growth in reticulocytes, expresses reticulocyte binding proteins [28] and requires a host Duffy blood group glycoprotein for invasion [29]. *P*. *vivax* infection causes accelerated remodelling of very young reticulocytes, a process that normally takes 24 hours in uninfected reticulocytes [30]. The rodent model malaria parasite, *P*. *berghei* is also 150 times more likely to invade reticulocytes and establish infection in the presence of equal numbers of mature erythrocytes and reticulocytes [31] and has therefore been long thought of as a suitable model for *P*. *vivax* blood stage biology [32].

Mature erythrocytes, comprising almost 98% of the circulating red blood cells, can be considered “simplified” cells; they are metabolically active but lack intracellular organelles found in the bone marrow erythroid precursors cells [33] and enucleated reticulocytes (maturing erythrocytes) that are present in peripheral circulation [34]. Reticulocytes undergo many changes after their release into the peripheral circulation as they mature and this is associated with a 20% decrease in total surface area and acquisition of a biconcave shape with consequent increase in shear membrane resistance, the progressive loss of organelles (mitochondria, ribosomes and lysosomes), the loss or reduced abundance of up to 30 membrane proteins, and decreased levels of membrane cholesterol [34,35]. This maturation process is associated with a general streamlining of cellular metabolism; mature erythrocytes are highly dependent on glycolysis [36] and the pentose phosphate pathway [37] for both energy and redox balance and lack many other pathways of carbon metabolism, such as citric acid cycle [38]. Reticulocytes are thus expected to contain a richer repertoire of carbon sources and other essential nutrients than mature erythrocytes which might be exploited or even required by reticulocyte preferent *Plasmodium* spp.

Limited comparative metabolomics of the erythroid lineage has been attempted before but focussed on sickle cell disease and cord blood reticulocyte physiology [39,40]. Therefore, in order to establish whether there are metabolic differences between reticulocytes and mature erythrocytes that could influence the tropism of different *Plasmodium* spp., we undertook a non-targeted, high coverage, comprehensive analysis of the metabolomes of these host cells. Comparison of the metabolomes of very young, uninfected rat and human reticulocytes and their mature erythrocyte counterparts revealed major biochemical differences that could be exploited by intracellular parasite stages. This was tested using reverse genetics to disrupt parasite metabolism and establish the broad ability of *P*. *berghei* to utilise the products of reticulocyte metabolism and (in part) explain differing profiles of drug susceptibility between parasites in mature erythrocyte and reticulocyte environments.

## Results

### The reticulocyte metabolome is more complex than that of the mature erythrocyte

**Fig 1:**
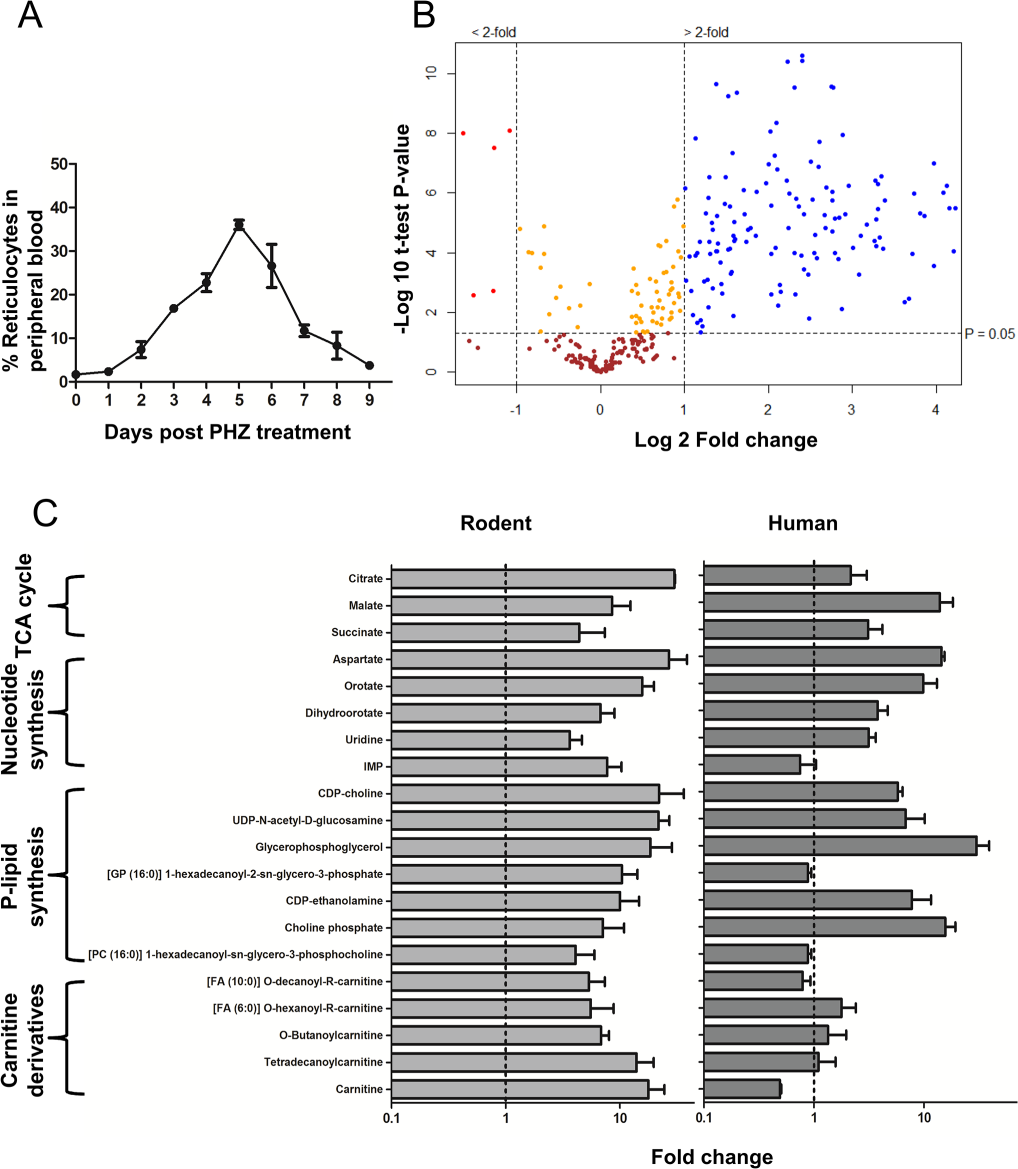
Comparison of Reticulocyte enriched Erythrocyte Population (REP) and wild type Erythrocyte Population (wtEP) reveals metabolite enrichment in rodent and human reticulocytes. A. Dynamics of reticulocyte enrichment in peripheral blood *in vivo* followed by Phenylhydrazine-HCl (phz) treatment of mice. Reticulocytes were harvested at day 5 post phz treatment. The error is given as the standard deviation (S.D.) of 3 independent biological replicates. B. Volcano plot showing distribution of putative metabolites according to their fold change in abundance in REP vs wtEP in rodent blood. All significant changes are represented above the broken horizontal line. Coloured dots indicate metabolites which are: Blue- significantly up-regulated, Red- significantly down-regulated, Yellow- significant but little change, Brown- non-significant. n = 3 independent biological replicates (with four internal technical replicates each). Significance tested by Welch’s T-test (α < 0.05). See Fig A-C in S1 Text and S1 Table in for the complete list of detected metabolites and their respective abundance fold changes. C. Representative metabolites up-regulated in reticulocytes compared to mature erythrocytes in human and rodent erythrocytes. Relative levels (peak intensities) are expressed as fold change observed in reticulocyte vs mature erythrocytes. Dotted line indicates no change and error bars indicate R.S.D. (Relative Standard Deviation) of peak intensities from reticulocyte samples multiplied to the fold change values from n = 3 independent biological replicates.

Induction of reticulocytosis was achieved through administration of phenylhydrazine-HCl (PHZ, 100 mg/kg body weight) to Wistar rats and cells were harvested when the percentage of reticulocytes in the peripheral blood reached a maximum at day 5 (~35% reticulocytes). This was monitored by FACS analysis using the reticulocyte surface marker transferrin receptor (CD71), which is lost as reticulocytes mature[35]. More than 90% of the 35% reticulocyte population generated by PHZ treatment were CD71-high at the time of harvest (Fig A-A in S1 Text) corresponding to the youngest of the four forms of reticulocytes that have been identified [39] and are from here on referred to as Reticulocyte enriched Erythrocyte Population (REP) Material was also collected for comparison with blood from non-enriched (~1% reticulocytes) animals- wild type Erythrocyte Population (wtEP) (Fig 1A). All samples were uniformly depleted of leucocytes.

Metabolite extracts of REP and wtEP were analysed in parallel by liquid chromatography mass spectrometry (LC-MS) and gas chromatography mass spectrometry (GC-MS), providing overlapping, as well as complementary coverage of the metabolomes of wtEP and REP. LC-MS data was processed using XCMS, MZMatch and IDEOM while GC-MS data was processed using PyMS matrix generation and Chemstation Electron Ionisation (EI) spectrum match analysis (described in detail in methods). A total of 333 metabolites were provisionally identified from a total of 4,560 mass features and peaks. The volcano plot in Fig 1B shows the distribution of abundance of detected metabolites in REP compared to wtEP. Almost half of all detected metabolites (147, ~45%) were found to be more than 2-fold more abundant in REP (with a p<0.05) (Fig 1B and A-C in S1 Text and S1 Table). Only 5 (~1%) metabolites were over 2-fold more abundant in wtEP than in REP (with p<0.05). The rest of the metabolites did not show a significant difference between REP and wtEP. Similar changes were observed when all mass features and peaks (~4,560 peaks) were included in the analyses. Specifically, of the ~4,230 unassigned mass features/peaks, 1,051 (~23%) were up-regulated and 91 peaks (~2%) down regulated in REP (Fig A-B in S1 Text). As the blood from reticulocytosis-induced rats still contained a major fraction of mature erythrocytes (1:2 final ratio of reticulocytes to mature erythrocytes) the level of metabolite enrichment in reticulocytes was actually much greater (column four, S1 Table). 20 representative metabolites up-regulated in rodent REP showed a similar ‘trend’ towards up-regulation in very young human reticulocytes grown *in vitro* from CD34+ stem cells [41] analysed using LC-MS (Fig 1C), except carnitine derivatives. All identified metabolites were charted on metabolic pathways known to exist in *Plasmodium* and mammalian host cell from biochemical studies [6,7,12,42,43] and genomic data [44], although it is expected that not all detected metabolites are endogenously synthesised, as plasma metabolites from other tissues, the microbiome, the diet and environment may also accumulate in erythrocytes.

### The reticulocyte metabolome reflects its ongoing developmental programme

Cell fractions from rodent REP contained elevated levels of glycolytic, pentose phosphate pathway and TCA cycle intermediates (S1 Table). The presence of the latter indicates that reticulocytes have a functional TCA cycle and associated intermediary carbon metabolism, consistent with the presence of a residual population of mitochondria in reticulocytes that are largely lost in mature erythrocytes [34]. Increases in the levels of intermediates of the purine and pyrimidine metabolic pathways in reticulocytes presumably originate either from biosynthesis in the preceding erythropoiesis stages or from catabolism of nucleic acid to their constituent nucleobases [45]. A number of intermediates of phospholipid metabolism were also elevated in reticulocytes compared to mature erythrocytes. Other notable changes included elevated levels of intermediates in glutathione and arginine metabolism in reticulocytes (S1 Table). In addition, many carnitine derivatives were found to be up-regulated in rodent (although interestingly not in human) reticulocytes which may relate to fatty acid catabolism by β-oxidation in the mitochondria or peroxisomes of these cells. Although decreased levels of carnitines have previously been found in human erythrocytes derived from normal subjects compared to individuals with Sickle-Cell (HbSS) disease [40], the procedures used for production of rodent reticulocytes (*in vivo*) and human reticulocytes (*in vitro*) cannot be ruled out as the reason for this difference observed between the two species as carnitine is produced in mammalian tissues (skeletal muscle, heart, liver, kidney, and brain) [46] a contributory factor missing in *in vitro* conditions. Almost 65% of the other metabolite ions detected in the HbSS study were also found to be present in erythrocytes in our analysis (S1 Table) and around 17% of metabolites detected in our analysis were also reported in erythrocytes in that study [40]. This difference in coverage could be due to the chromatographic and detection methods which differ between the analyses.

Taken together these data demonstrate that the reticulocyte contains elevated levels of many metabolites that could potentially be scavenged by the invading malaria parasite. Furthermore, there was a marked overlap in metabolic pathways observed in the reticulocyte and those predicted in the parasite [43,44]. Common pathways might therefore be uniquely dispensable to *Plasmodium* during its growth in the reticulocyte compared with that in mature erythrocytes. To test this hypothesis, we used reverse genetics to target several metabolic pathways in intermediary metabolism and pyrimidine biosynthesis in *P*. *berghei* whose intermediates were significantly up-regulated in reticulocytes.

### Features of intermediary carbon metabolism are dispensable in asexual blood stage *P*. *berghei*

**Fig 2:**
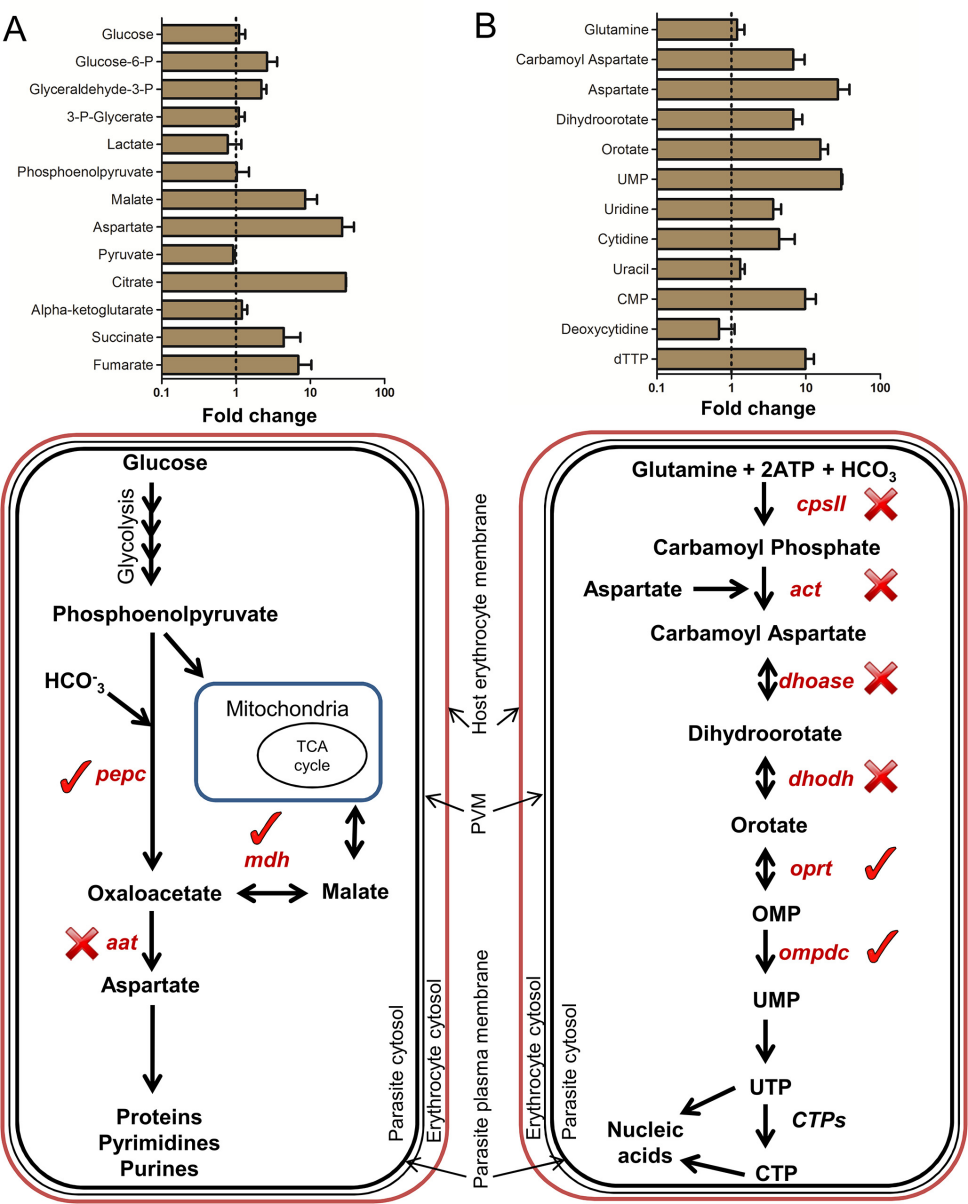
Metabolites of intermediary carbon metabolism (ICM) and pyrimidine biosynthesis are up-regulated in reticulocytes. A. Top panel: Fold change of relative levels (peak intensities) of metabolites of carbon metabolism in rodent REP compared to wtEP. Dotted line indicates no change and error bars indicate R.S.D. (Relative Standard Deviation) of peak intensities from reticulocyte samples multiplied to the fold change values from n = 3 independent biological replicates. Bottom panel: Schematic representation of intermediary carbon metabolism (ICM) in *Plasmodium* cytosol. Genes marked with (✓) were deleted in *P*. *berghei* blood stages and the ones marked with (✕) could not be deleted even after repeated attempts. *pepc*: Phosphoenolpyruvate Carboxylase (PBANKA_101790), *mdh*: Malate Dehydrogenase (PBANKA_111770), *aat*: Aspartate Amino Transferase (PBANKA_030230). B. Top panel: Fold change of relative levels (peak intensities) of metabolites of pyrimidine biosynthesis in rodent REP compared to wtEP. Dotted line indicates no change and error bars indicate R.S.D. (Relative Standard Deviation) of peak intensities from reticulocyte samples multiplied to the fold change values from n = 3 independent biological replicates. Bottom panel: Schematic representation of pyrimidine biosynthesis pathway in *Plasmodium* cytosol. Genes marked with (✓) were deleted in *P*. *berghei* blood stages and the ones marked with (✕) could not be deleted even after repeated attempts. *cpsII*: Carbamoyl phosphate synthetase II (PBANKA_140670), *act*: Aspartate carbamoyltransferase (PBANKA_135770), *dhoase*: Dihydroorotase (PBANKA_133610), *dhodh*: Dihydroorotate dehydrogenase (PBANKA_010210), *oprt*: Orotate phosphoribosyltransferase (PBANKA_111240), *ompdc*: Orotidine 5′-monophosphate decarboxylase (PBANKA_050740). (Also see Fig B in S1 Text for gene deletion strategy and confirmation)

Asexual red blood cell stages of *Plasmodium* spp. catabolize glucose via the intermediary carbon metabolic pathways depicted in Fig 2A and express the cytosolic enzymes, phosphoenolpyruvate carboxylase (*pepc* PBANKA_101790), malate dehydrogenase (*mdh* PBANKA_111770) and aspartate amino transferase (*aat* PBANKA_030230). De novo synthesis of aspartate is likely to be important for nucleic acid synthesis as this amino acid is utilised in both purine salvage and as a carbon skeleton in pyrimidine biosynthesis [47] and inhibition of *aat* has been shown to be lethal to *P*. *falciparum* [48]. Malate produced by these pathways either enters mitochondria to participate in the TCA cycle or is excreted [7,42].

**Fig 3:**
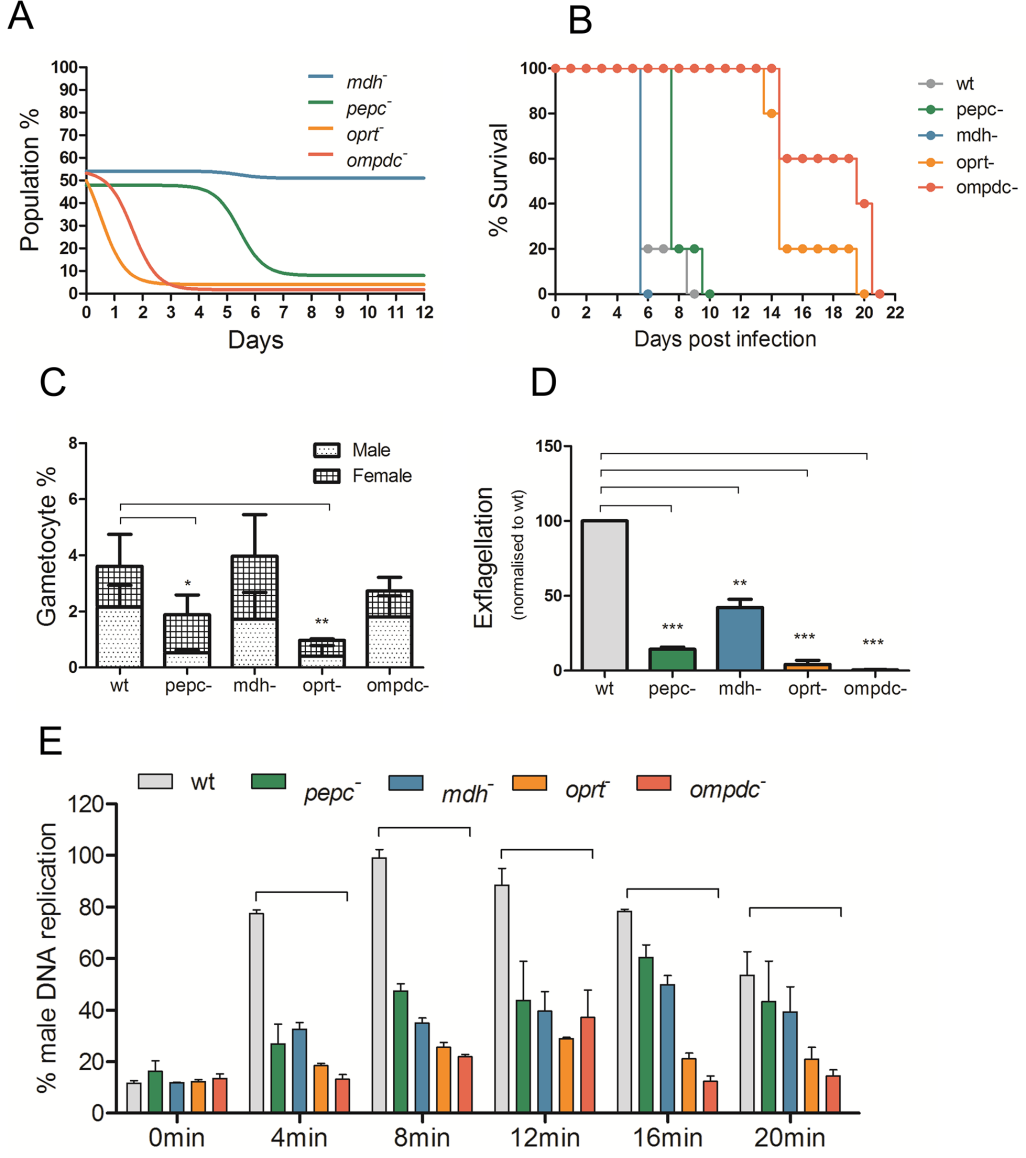
Phenotypic analyses of blood stage mutant *P*. *berghei* parasites. A. *in vivo* growth assay of mutants in mixed infections in competition with wild type parasites over 12 days. Coloured lines represent non-linear fit of percentage of mutant parasites in total parasite population. Data representative of n = 3 independent biological replicates. (Also see Fig C- A, B and C in S1 Text.) B. Lethality experiment in C57/B6 mice by wt and mutant *P*. *berghei* parasites. 10^4^ parasites were injected intra-peritoneally in mice (n = 5) on day 0 and they were monitored for 21 days. The mice were culled humanely when they showed severe malaria pathology. All mutant parasites were found to be lethal to mice. C. Gametocyte conversions during blood stages in mutant *P*. *berghei* parasites over 5 days post infection. Data from 2 independent observed gametocyte conversion experiments are shown ± S.D. Gametocyte conversion was observed using a wt parent line which expresses GFP in male gametocytes and RFP in female gametocytes (RMgm-164). *P*. *berghei* mutants were generated in the same genetic background and analysed using FACS determining the number of gametocytes in infected blood. P-values: *p<0.05, **p<0.005, ***p<0.0005, paired two tailed t-test. D. Exflagellation (male gamete formation) in mutant *P*. *berghei* parasites normalised to wt in *in vitro* activation assay. The error is given as the SD of n = 3 independent biological replicates. P-values: **p<0.005, ***p<0.0005, paired two tailed t-test. E. Determination of DNA content of male gametocytes over 20 minutes post activation by FACS analysis in mutant *P*. *berghei* parasites normalised to wt. DNA content was determined in Hoechst-33258-stained MACS purified gametocytes. Before activation (0minutes) males show low DNA content with increasing amounts post activation reaching maximum levels between 8 to 12 minutes in wt. Data from 3 independent biological replicates are given ± S.D. P-values: **p<0.005, ***p<0.0005, unpaired two tailed t-test (also see Fig C- D in S1 Text).

**Fig 4:**
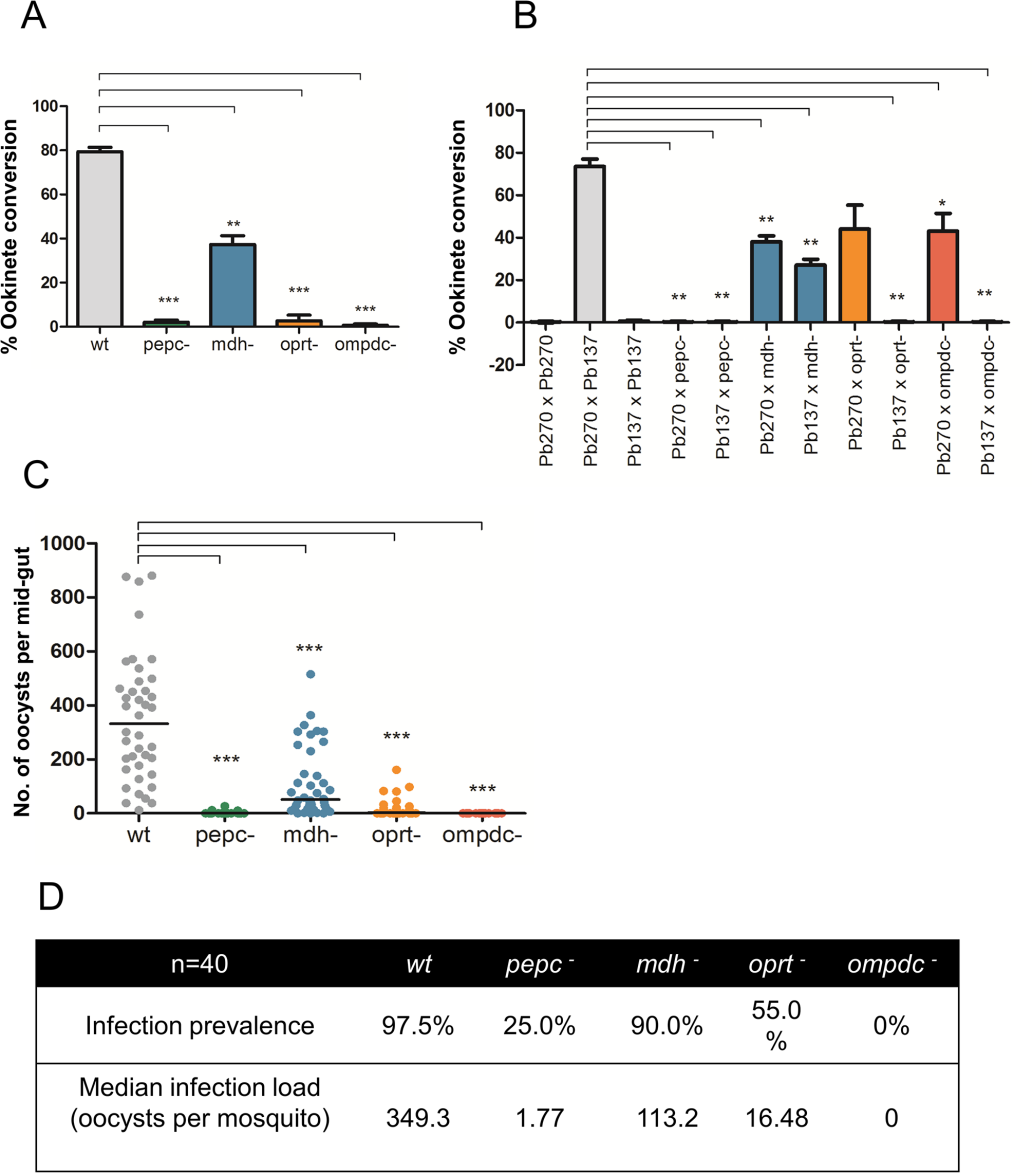
Mosquito stage development of *P*. *berghei* mutant parasites (also see Fig D in S1 Text). A. *in vitro* ookinete conversion of mutant *P*. *berghei* parasites as compared to wt. The error is given as the S.D. of n = 3 independent biological replicates. P-values: **p<0.005, ***p<0.0005, unpaired two tailed t-test. B. *in vitro* ookinete conversion assay to measure fertility of mutant *P*. *berghei* gametocytes. Fertility of mutant *P*. *berghei* gametocytes was analysed by their capacity to form ookinetes by crossing gametes with RMgm-348 (Pb270, *p47* ^*-*^) which produces viable male gametes but non-viable female gametes and RMgm-15 (Pb137, *p48/45* ^*-*^) which produces viable female gametes but non-viable male gametes. The error is given as the S.D. of n = 2 independent biological replicates. P-values: *p<0.05, **p<0.005, unpaired two tailed t-test. C. Number of mature oocysts at day 14 post infected blood feed in mosquito mid guts. n = 40 mosquitoes cumulative of two independent biological replicates. ***p<0.0005, unpaired two tailed t-test. D. Infection prevalence (percentage of observed mosquitoes found to be infected) and infection load (median of number of oocysts found per mosquito) in mutant *P*.*berghei* parasites compared to wt.

Metabolites involved in TCA cycle and intermediary carbon metabolism (ICM), including malate and aspartate, were found to be substantially higher in REP compared to wtEP (Fig 2A). The elevated levels of these intermediates may possibly explain the previous observation that disruption of the TCA cycle in *P*. *berghei* blood stages through deletion of flavoprotein (Fp) subunit of the succinate dehydrogenase, *pbsdha* (PBANKA_051820), had little effect on parasite viability in blood stage forms, although ookinete development was impaired [49]. To further explore the possibility that *P*. *berghei* has potential access to the anapleurotic substrates of reticulocyte ICM, attempts were made to delete *pepc*, *mdh* and *aat* in *P*. *berghei* and assess the importance of these parasite enzymes throughout the life cycle (Fig 2A). *P*. *berghei* mutants lacking both *pepc* and *mdh* were generated (Fig B in S1 Text), while deletion of *aat* proved refractory. Both the *pepc* ^*-*^ and *mdh* ^*-*^ mutant parasites caused severe cerebral malaria in CD57/B6 mouse model with similar dynamics to wt parasites (Fig 3B). Interestingly, the growth of the *pepc* ^*-*^ mutant was compromised compared to wild type parasites, as the *pepc* ^*-*^ mutant, but not the *mdh* ^*-*^ mutant was overgrown by the wt parasite in an *in vivo* sensitive single host competitive growth assay (Fig 3A and C-A in S1 Text). The number of merozoites observed in mature schizont stages in both *pepc* ^*-*^ (17.02 ± 1.8) and *mdh* ^*-*^ (17.41 ± 1.7) mutants are similar to wt (17.4±1.8) (Fig C-C in S1 Text). Scrutiny of the growth phenotype detected in the *pepc* ^*-*^ mutants showed that they have a prolonged asexual cycle (4 h longer than wt) (p<0.05) (Fig C-B in S1 Text). The number of gametocytes formed in blood stages was also reduced in *pepc* ^*-*^ mutants by almost 50% but unaffected in *mdh* ^*-*^ (p>0.05) (Fig 3C) with no notable difference in male to female ratio in either mutant. Further phenotypic analyses showed reduction of exflagellation (*pepc* ^*-*^ mutants 84% less than wt, p<0.0005; *mdh* ^*-*^ mutants 56% less than wt, p<0.005) (Fig 3D). DNA replication in male gametocytes as observed by FACS analysis was reduced by 50% compared to wt at the 8 minute time point and further delayed taking up to 16 minutes to complete (Fig C-E and C-D in S1 Text). Ookinete development in *in vitro* cultures of *pepc* ^*-*^ mutants was also severely affected while in *mdh* ^*-*^ mutants, ookinetes were formed but the number was reduced by about 50% compared to wt (Fig 4A). To determine if this defect was sex specific, crosses of *pepc* ^*-*^ and *mdh* ^*-*^ were performed with *P*. *berghei* lines RMgm-348 (Pb270, *p47* ^-^) which produces viable male gametes but non-viable female gametes and RMgm-15 (Pb137, *p48/45* ^*-*^) which produces viable female gametes but non-viable male gametes [50]. Mutants of *pepc* ^*-*^ were found to produce severely reduced numbers of ookinetes in either cross suggesting that gametes of both genders are affected and that the activity of the protein is essential for viable gamete formation. This was not the case for *mdh* ^*-*^ mutants where although crossing experiments showed that lack of MDH protein affected both genders, they mimicked the parental phenotype producing 50% fewer mature ookinetes (Fig 4B). The *pepc* ^*-*^ parasites were defective in development within the mosquitoes, forming small numbers of oocysts in mosquito midguts and no salivary gland sporozoites. However, parasites lacking *mdh* could complete transmission through the mosquito and infect mice generating blood stage asexual forms in 48–72 hours similar to wt despite producing reduced numbers of oocysts when compared to wt (Fig 4C and 4D and D-A and D-B in S1 Text). Overall, these results suggest that two key enzymes in *P*. *berghei* ICM are at least partially redundant during stages of infection in which the parasites resides primarily in reticulocytes, but that they become essential as parasite differentiates and proliferates within other host or vector cell types.

### Pyrimidine biosynthesis can be partially disrupted in reticulocyte-preferent *P*. *berghei*

*Plasmodium* spp. are heavily dependent on nucleic acid synthesis during blood stage asexual growth and either salvage (i.e. purines) or synthesize (i.e. pyrimidines) the requisite bases. A schematic representation of the pyrimidine biosynthesis pathway is given in Fig 2B. Five out of six enzymes of this pathway have been shown to be essential for *P*. *falciparum* growth in standard *in vitro* cultures, based on pharmacological studies [51]. Interestingly, most of these inhibitors are markedly less potent in the *in vivo P*. *berghei* model, a feature that has been attributed to reduced bio-availability of inhibitors in mice or apparent differences in target enzyme structures [52,53]. However, increased resistance to pyrimidine biosynthetic inhibitors could also reflect higher concentrations of pyrimidine precursors (bar glutamine) in the reticulocyte population selectively colonized by this species (Fig 2B) [16,51]. To investigate this possibility we attempted to delete in *P*. *berghei* 6 genes encoding enzymes involved in pyrimidine biosynthesis; carbamoyl phosphate synthetase II (*cpsII*) (PBANKA_140670), aspartate carbamoyltransferase (*act*) (PBANKA_135770), dihydroorotase (*dhoase*) (PBANKA_133610), dihydroorotate dehydrogenase (*dhodh*) (PBANKA_010210), orotate phosphoribosyltransferase (*oprt*) (PBANKA_111240) and orotidine 5′-monophosphate decarboxylase (*ompdc*) (PBANKA_050740). While the first four enzymes in this pathway were refractory to deletion, the last two enzymes in pyrimidine biosynthesis, orotate phosphoribosyltransferase (*oprt*) and orotidine *5′*-monophosphate decarboxylase (*ompdc*) could be deleted (Fig B in S1 Text). The *oprt* ^*-*^ and *ompdc* ^*-*^ mutant parasites grew slowly (asexual cycle prolonged by approximately 4–5 hours compared to wt (p<0.05)), were rapidly outgrown in a competition growth assay with wt parasites (Fig 3A) and based on gray value^-1^ of staining intensity as observed by Giemsa staining (p<0.0005), seem to invade very young reticulocytes (Fig C-E in S1 Text). However, these infected reticulocytes could not be classified as CD71-high possibly due to the accelerated loss of the CD71 as observed with *P*. *vivax* infected reticulocytes [30]. Furthermore, both *oprt* ^*-*^ mutants (15.9 ± 2.0, p<0.0005) and *ompdc* ^*-*^ mutants (15.2 ± 2.5, p<0.0005) were found to generate, on average, significantly fewer merozoites than wt parasites (17.5 ± 1.8) per schizont (counted after completion of asexual cycle) (Fig C-C in S1 Text) and the asexual parasites also took longer to mature to schizonts (Fig C-B in S1 Text). Both mutants showed altered lethality in the C57/B6 mouse model as the mice infected with the mutants did not manifest the symptoms of experimental cerebral malaria (ECM) but died between days 14–20 as a result of severe anaemia and hyperparasitemia (Fig 3B). The process of transmission was also affected by the loss of *ompdc* and *oprt*. Gametocytemia was significantly reduced only in *oprt* ^*-*^ parasites (Fig 3C) but no change was seen in male- female ratio. Exflagellation (the production of mature male gametes) was found to be severely affected in *oprt* ^*-*^ and completely blocked in *ompdc* ^*-*^ parasites (Fig 3D) and DNA replication during male gametogenesis was severely reduced (Fig 3E). Consistent with the defects in male gametogenesis, very few ookinetes were formed in *in vitro* cultures in *oprt* ^*-*^ parasites and no ookinetes were observed in *ompdc* ^*-*^ (Fig 4A). Genetic crosses of *oprt* ^*-*^ and *ompdc* ^*-*^ mutants were performed as above with *P*. *berghei* lines RMgm-348 and RMgm-15 which showed that viable male gametes (from RMgm-348) were able to rescue the ookinete conversion defect in both mutant lines suggesting that formation of male gametes is impaired in both *oprt* ^*-*^ and *ompdc* ^*-*^ mutant parasites while female gametes remain unaffected (Fig 4B). Infectivity to the mosquito was significantly reduced in *oprt* ^*-*^ and completely blocked in *ompdc* ^*-*^ mutants as seen by observing oocysts in infected mosquito midguts and salivary gland sporozoites (Fig 4C and 4D and D-C and D-D in S1 Text) and infection to naïve mice was found to be completely blocked. However, when ookinetes from *p47* ^*-*^ x *oprt* ^-^ or *ompdc* ^-^ crosses were fed to mosquitoes, they failed to develop into mature oocysts (Fig E in S1 Text) hence, did not complete sporogony indicating that lack of both *oprt* and *ompdc* in the female lineage results in an allelic insufficiency in a growing oocyst.

**Fig 5:**
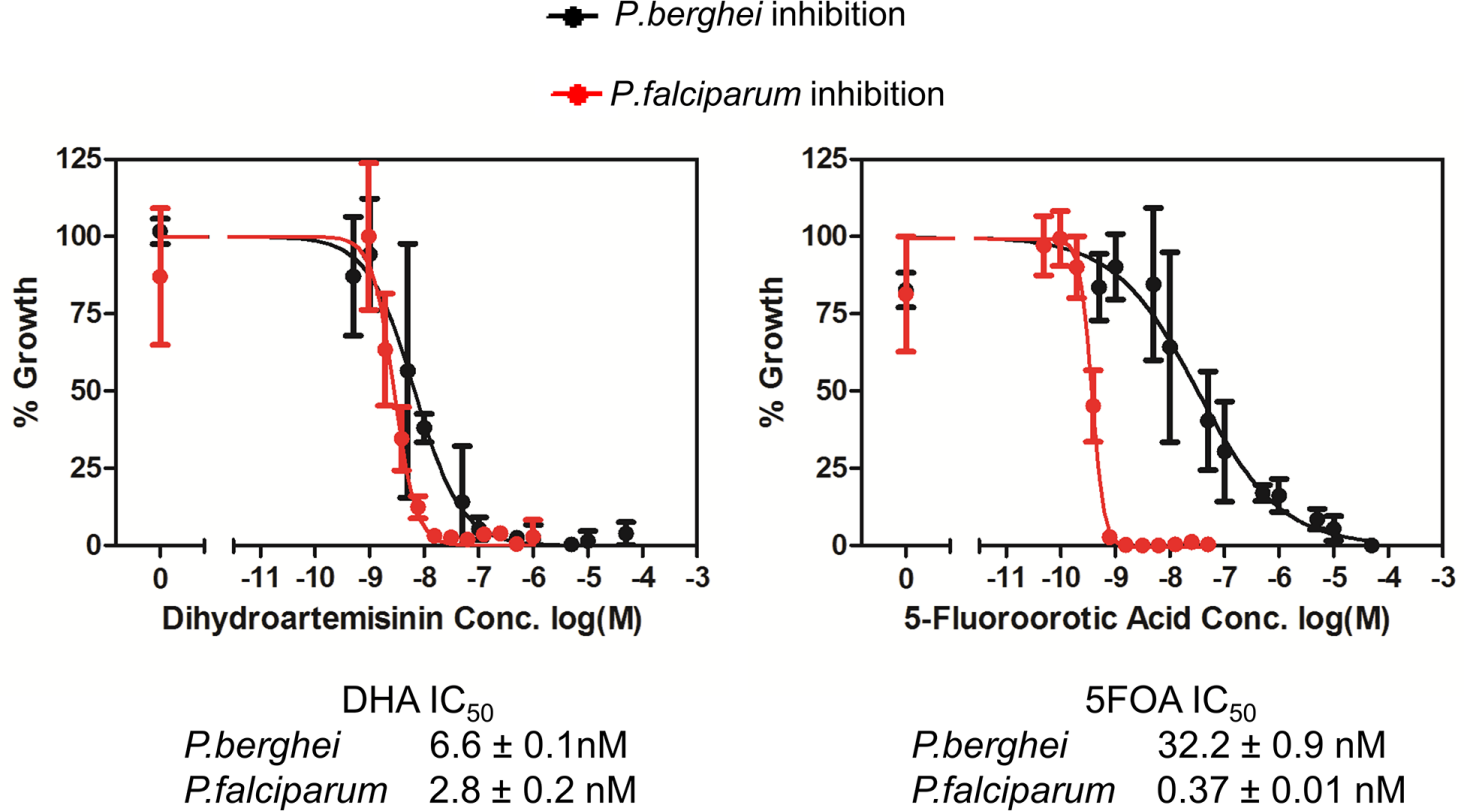
*P*. *berghei* and *P*. *falciparum* inhibition by dihydroartemisinin (DHA) and 5-fluoroorotic acid (5FOA) *in vitro*. Error bars indicate S.D. from n = 3 biological replicates.

We also tested the effect of a previously published inhibitor of pyrimidine biosynthesis 5-fluoroorotate (5FOA) [54] on asexual growth of both *P*. *falciparum* and *P*. *berghei*. The comparisons were carried out *in vitro* to prevent bioavailability of the inhibitor confounding *in vivo* data in mice. We tested the activity and found that the IC_50_ value of 5FOA *in vitro* was almost 90-fold higher in *P*. *berghei* (32.2 ± 0.9 nM) compared to *P*. *falciparum* (0.37 ± 0.01 nM) (Fig 5). A dihydroartemisinin control showed no major difference in inhibition between *P*. *berghei* (6.6 ± 0.1 nM) and *P*. *falciparum* (2.8 ± 0.2 nM). These data strongly suggest that *P*. *berghei* can access pyrimidine precursors from the reticulocyte and are consistent with a role of host cell metabolism in the differential activity of 5FOA, although differences in sensitivity of *P*. *falciparum* [55] and *P*. *berghei* [56] thymidylate synthase or differences in drug uptake could also contribute to the differential lethality.

## Discussion

These metabolomics analyses clearly showed that reticulocytes have a much more complex metabolome than mature erythrocytes, adding to previously well documented changes that occur in both organelle complement and protein expression levels during reticulocyte maturation in peripheral circulation [34,35]. Key metabolic processes that were found to be elevated in REP but absent or highly reduced in wtEP included the TCA cycle and associated intermediary carbon metabolism, nucleic acid metabolism, phospholipid metabolism, fatty acid catabolism and glutathione metabolism. The down-regulation of these host pathways in wtEP may explain why several of the corresponding pathways in asexual blood stages of *P*. *falciparum* appear to be essential *in vitro*. Conversely, we predicted that malaria parasites infectious to both humans (*P*. *vivax*) and rodents (*P*. *berghei*) which exhibit a strong tropism for reticulocytes rather than mature erythrocytes may be more tolerant to loss of key metabolic pathways because of redundancy with host pathways. Our data strongly suggest that reticulocytes do indeed provide a highly enriched host cell niche for *Plasmodium*, with important implications for drug discovery strategies. Although the reticulocyte offers a very different nutrient resource compared to the mature erythrocyte, there has been apparently little change in the metabolic capacity of *P*. *vivax* and *P*. *berghei*. Our *in silico* comparison (S3 Table) reveals that *P*. *vivax* and *P*. *berghei* have 98% and 97% orthology, respectively, to *P*. *falciparum* genes annotated with metabolic pathway information (434 genes) on PlasmoDB.

### The enriched reticulocyte metabolome supports growth of metabolically compromised *P*. *berghei*

Although a small number of metabolomics studies have been undertaken on erythrocytes, including a comparison of normal and HbSS erythrocytes, the number and range of metabolites detected in these studies were relatively small (20–90) [39,40]. Here we used complementary LC-MS and GS-MS analytical platforms to maximise coverage, generating the most comprehensive coverage of REP and wtEP undertaken to date (333 metabolites). These studies revealed a much higher degree of metabolic complexity in reticulocytes compared to mature erythrocytes covering nearly all major pathways in central carbon metabolism. Whilst glycolysis is the main pathway for carbon metabolism in erythrocytes [36], both human [57] and rodent [58] erythrocytes retain a residual proteomic signature of TCA cycle and ICM enzymes and our metabolomics data suggests that these pathways are much more active in reticulocytes, leading to elevated levels of TCA intermediates (including citrate, malate) and ICM products (*e*.*g*. aspartate). The functional significance of increased metabolic complexity in reticulocytes was subsequently tested by generating *P*. *berghei* mutants with specific defects in metabolism showing that the increased availability of complementary metabolites in reticulocytes can explain the non-essential nature of the *P*. *berghei pepc* and *mdh* genes, which are involved in regulating intracellular levels of oxaloacetate and malate. In contrast, PEPC is essential for normal intra-erythrocytic survival of *P*. *falciparum in vitro*, although this can be bypassed by malate supplementation of *P*. *falciparum* infected mature erythrocytes [42]. It should be noted that whilst the *P*. *berghei pepc* ^-^ mutant retained its virulence, it still showed a significant growth defect compared with wild type parasites (similar to the *P*. *falciparum* mutant [42]) resulting at least in part from a prolongation of the asexual blood stage cycle as revealed by our sensitive single host competitive growth assay. It would be interesting to use this assay to compare asexual growth dynamics of other available metabolic mutants such as the *pbsdha* ^*-*^ with wild type which might reveal additional defects to those reported [49]. The *P*. *berghei pepc* ^*-*^ mutant also failed to complete transmission through mosquitoes as a result of defects in gametocyte production, male gamete formation, female gamete viability resulting in trace oocyst formation and failure to enter sporogony, which extends our understanding of the importance of this metabolic enzyme for parasite development beyond the asexual blood stages previously investigated [42]. A possible explanation for this phenotype is that the *pepc* ^*-*^ mutant is unable to by-pass the need for *de novo* synthesized aspartate for nucleotide biosynthesis by salvage from different host cells during its sexual and asexual life cycle (Fig 2A). The demonstration of *pbpepc* ^-^ growth in reticulocytes suggests that the equivalent *P*. *falciparum* mutant might be a suitable candidate for an attenuated slow growing parasite vaccine that would permit generation of significant anti-parasitic immune responses.

In line with this suggestion, we were unable to delete *pbaat* which is also required for *de novo* synthesis of aspartate. The essential nature of *pbaat* suggests that either the apparently higher levels of aspartate in reticulocytes are insufficient to meet the demands of a growing asexual stage parasite or that, as in *P*. *falciparum* intra-erythrocytic stages, *P*. *berghei* is not readily able to access host cytoplasmic pools of aspartate [59]. Production of aspartate in *Plasmodium pepc* ^-^ mutants can still be achieved through generation of the oxaloacetic acid precursor by mitochondrial malate: quinone oxidoreductase (MQO) or the reverse reaction of cytosolic MDH. However, this is apparently a suboptimal solution for the *pepc* ^-^ parasite resulting in slow growth in the blood stage and failure to develop in the mosquito. *Plasmodium* AAT can also generate methionine from aspartate, glutamate and other amino acids which can act as effective amino group donors [60] and regulate glutamine/glutamate metabolism. These functions may not be rescued by simple aspartate salvage from the host and further support the essentiality of *aat* as a key enzyme for the parasite and a possible drug target.

The *P*. *falciparum* gene encoding MDH has proved refractory to deletion under any circumstances so far, suggesting that it is essential for these parasites. In marked contrast, the *P*. *berghei* mutants lacking *pbmdh* were readily generated, suggesting that this species may scavenge reticulocyte pools of malate or other intermediates in the TCA cycle. The *pbmdh* mutant exhibited a very modest growth phenotype and was able to develop into mosquito infective stages, although it produced 30% fewer oocysts than wt parasites. The continued viability of the *pbmdh* ^*-*^ mutants during transmission in the absence of reticulocyte-based compensatory sources of the metabolite can be explained by continued TCA derived production of malate and NADH+ H^+^ reducing equivalents given the increased flux through the TCA metabolism in gametocytes and probably later sexual stages [7,12]. Conditional silencing or disruption of *pfmdh* or degradation of PfMDH in mature gametocytes or later stages of *P*. *falciparum* would establish if MDH is required for transmission of the human parasite and that the essential nature of this enzyme is merely blood stage specific.

### *P*. *berghei* is partially capable of pyrimidine salvage in highly immature reticulocytes

*Plasmodium* spp. salvage their purine requirements from the host cell, but retain the ability to synthesise pyrimidines [61]. Purine nucleosides are taken up by the parasite PfNT1 and other, as yet, unidentified AMP transporters [62] after they are delivered to the parasitophorous vacuole via the action of erythrocyte nucleoside transporters [51,63] and a non-selective transport process [61,64]. In contrast, while other Apicomplexans (i.e. *Cryptosporidium* spp., *Toxoplasma* spp.) retain the capacity to salvage pyrimidines [16], *Plasmodium* spp. are thought to lack enzymes required for host pyrimidine salvage [44]. Although, *Plasmodium* proteins have been implicated in transporting some pyrimidine precursors [65,66], presumably due to very limited availability of pyrimidines in the host cell, *Plasmodium* parasites have been thought to be completely dependent on *de novo* pyrimidine synthesis for growth in asexual stages [51].

The survival of both *oprt* ^-^and *ompdc-* mutants could be the result of two possibilities that are not mutually exclusive. The first possibility is that the mutants directly utilize reticulocyte pools of pyrimidines which are nonetheless limiting leading to a reduction in number of merozoites produced. Alternatively, mutant parasites could synthesize orotate which is secreted into the host cytoplasm and converted to UMP by host UMP synthase before being salvaged. Both outcomes require transport of nucleosides or nucleotides from the host cytoplasm to the parasite and how this is achieved is not clear. Both pyrimidine biosynthesis mutants survive only in the youngest reticulocytes which might reflect either adequacy of supply of host UMP (or derivatives) or the capacity of the youngest reticulocytes to convert parasite-derived orotate. Indeed enzymes involved in the later stages of pyrimidine biosynthesis, nucleoside diphosphate kinase B, CTP synthase and ribonucleotide reductase large subunit have been identified in rodent and human erythrocytes [57,58]. The possibility that host pyrimidine enzymes may have redundant functions with the parasite enzymes catalysing late steps in pyrimidine biosynthesis is supported by the apparent essentiality of the *P*. *berghei* genes encoding the first four steps of pyrimidine biosynthesis. A simplified illustration of life cycle stages of *P*. *berghei* development showing the characteristics of mutant parasites at various points in the life cycle is shown in Fig G in S1 Text.

### Glutathione biosynthesis is elevated in reticulocytes

The REP metabolome also explains other species-specific differences between *P*. *berghei* and *P*. *falciparum*. Glutathione biosynthesis occurs in erythrocytes [67] and the enzymes for this pathway have been shown to be present in both human [57] and rodent [58] erythrocytes. *Plasmodium* employs its own glutathione redox system [68] to counter oxidative stress (Fig F-A in S1 Text). Both *ɣ*-glutamylcysteine synthetase (*ɣ-gcs*) and glutathione synthetase (*gs*) are essential for parasite survival in *P*. *falciparum* [69] yet *ɣ-gcs* and glutathione reductase (*gr*) can be deleted in *P*. *berghei* and intra-erythrocytic asexual growth is unaffected although mosquito stage development is arrested at the oocyst stage [70,71]. The REP and wtEP metabolomes demonstrated that the levels of glutathione synthesis intermediates were higher in reticulocytes than in mature erythrocytes (Fig F-B in S1 Text) providing a mechanistic explanation for the normal growth of *P*. *berghei ɣ-gcs* and *gr* mutant asexual stages in reticulocytes. Also, the inhibitor of ɣ-*gcs*, buthionine sulphoximine (BSO) inhibits *P*. *falciparum* growth with an IC_50_ value of ~60 μM [69] yet concentrations as high as 500 μM BSO *in vitro* had no inhibitory effect on *P*. *berghei* parasites in *in vitro* cultures (Fig F-C in S1 Text) consistent with the reticulocyte mediated rescue of chemical disruption of the glutathione synthesis pathway in *P*. *berghei*, although it has not been investigated whether there is a difference in BSO sensitivity against the mouse or *P*. *berghei ɣ-gcs* compared to *P*.*falciparum* or human enzymes.

### Host cell metabolism can ameliorate the impact of different drug treatments

Enzymes involved in *Plasmodium* intermediary carbon metabolism [12,42] and pyrimidine biosynthesis [51] are considered attractive targets for drug development. The metabolome surveys and drug inhibition data presented here suggest that caution should be used before extrapolating conclusions regarding gene essentiality in reticulocyte preferent parasites such as *P*. *berghei* as part of any drug discovery pathway that has been based initially upon screens in mature erythrocytes. Bioavailability in mouse models and/or drug penetration into the reticulocyte and difference in target enzyme structures between species have been proposed as reasons for the relative ineffectiveness of drugs when tested *in vivo* using *P*. *berghei* [52,53]. An alternative view is that the reticulocyte metabolome (at least in part) provides a reservoir of metabolites downstream of the point of action of a drug rendering the drug less effective. This has a number of consequences:

1. Good drug candidates for *P*. *falciparum* might be (already have been) eliminated from further development due to adverse and misleading data from *in vivo P*. *berghei* testing.
2. Alternative mature erythrocyte preferent rodent parasites (e.g. *P*. *yoelii* YM) [72] might provide a more accurate *in vivo* model in which to test drug candidates for *P*. *falciparum* malaria.
3. *P*. *berghei* could provide an accurate *in vivo* model for the development of drugs against reticulocyte preferent parasites such as *P*. *vivax*, and this warrants further investigation.
4. Recrudescence of *P*. *falciparum* infection during treatment with anti-malarials that target parasite metabolism (anti-metabolites) might result from their survival in a ‘protective’ reticulocyte niche, the existence of which cannot be predicted by standard *in vitro* cultures.
5. Reticulocyte resident parasites exposed to anti-metabolites at levels determined by *in vitro* culture in mature erythrocytes would be sub-optimal with the subsequent risk of being more conducive for the selection of drug resistant progeny.

## Materials and Methods

### Metabolomics of rodent erythrocytes

Rodent reticulocyte enrichment was done in rats by administering phenylhydrazine-HCl dissolved in 0.9% NaCl (w/v) at a dose of 100 mg/kg body weight and collecting reticulocyte enriched peripheral blood on day 5 post injection. Metabolite extraction was done as using chloroform/methanol/water (1:3:1 v/v) and samples were analysed using LC-MS and GC-MS. See S1 supplementary materials and methods for details.

### Metabolomics of human CD34+ stem cell grown erythrocytes

CD34+ cells obtained from blood from human volunteers were cultured in a three-stage protocol based on the methods of [41]. Cultured reticulocytes and mature erythrocytes from matching donors were used for metabolite extraction with chloroform/methanol/water (1:3:1 v/v) and samples were analyzed using LC-MS. See S1 supplementary materials and methods for details.

### *P*. *berghei* methods

Infection of laboratory mice, asexual culture of *P*. *berghei* stages and generation of knockout parasites was done as before [73]. Asexual growth competition assay was done by mixing wt and mutant parasites expressing different fluorescent markers and injecting them intravenously into recipient mice and monitoring the growth of the two populations by flow cytometry as done before [74]. Lethality of mutant *P*. *berghei* parasites was checked by injecting infected RBCs (10^4^) into C57/B6 mice and monitoring parasitaemia, disease pathology and mortality over 21 days. Gametocyte conversion was monitored by flow cytometry in mutants generated in parent line (820cl1m1cl1) expressing GFP in male gametocytes and RFP in female gametocytes [75]. DNA quantification during exflagellation was also monitored by flow cytometry in mutant *P*. *berghei* parasites. Development of ookinetes in wild type, mutants and sexual crosses was observed in standard in vitro cultures maintained at 21°C. Mosquito transmission experiments were done in 5–8 days old mosquitoes used for infected blood feeds at 21°C and monitored for oocyst and sporozoite development. See S1 supplementary materials and methods for details.

### Determination of IC_50_ value of inhibitors *in vitro*

Inhibitors were used to perform *in vitro* drug susceptibility tests in standard cultures of synchronized *P*. *berghei* and *P*. *falciparum* blood stages. For testing *P*. *berghei* inhibiton, inhibitors were used at increasing concentrations to culture ring stage *P*. *berghei* for 24 hours and parasite development to schizont stage was analyzed by flow cytometry after staining iRBCs with DNA-specific dye Hoechst-33258. *P*. *falciparum* 3D7 strain was used for determining IC_50_ values of inhibitors in *in vitro* cultures by measuring ^3^H-Hypoxanthine incorporation in the presence of inhibitors in increasing concentrations. See S1 supplementary materials and methods for details.

### Ethics statement

All animal work was approved by the University of Glasgow’s Animal Welfare and Ethical Review Body and by the UK’s Home Office (PPL 60/4443). The animal care and use protocol complied with the UK Animals (Scientific Procedures) Act 1986 as amended in 2012 and with European Directive 2010/63/EU on the Protection of Animals Used for Scientific Purposes. Blood from human volunteers was supplied by the Australian Red Cross Blood Service and experiments were approved by the Walter and Eliza Hall Institute Human Research Ethics Committee, Australia. As a part of standard Australian Red Cross Blood Service practice, blood was collected from healthy donors who were informed about this study and potential risks to them and gave written consent when they donated blood.

## Supporting Information

S1 Text Fig A. A. Characterisation of the enriched reticulocyte population induced by Phenylhydrazine-HCl (PHZ) by FACS analysis on day 5 post PHZ administration. Top panel shows Ter119-FITC staining in RBCs which stains all erythroid cells. Bottom panels show CD71-APC staining in RBCs which stains only reticulocytes (~35%) and the population of CD71-high reticulocytes is almost 90% indicating that the majority of reticulocytes are very young. B. Volcano plot showing the distribution of abundance of all ~4560 peaks detected across both LC-MS and GC-MS platforms in Reticulocyte enriched Erythrocyte Population (REP) as compared to wild type Erythrocyte Population (wtEP) in rodent blood. All significant changes are represented above the broken horizontal line. Coloured dots indicate peaks which are: Blue- significantly up-regulated, Red- significantly down-regulated, Yellow- significant but little change, Brown- non-significant. n = 3 independent biological replicates (with four internal technical replicates each). Significance tested by Welch’s T-test (< 0.05). C. Fold change of metabolite abundance in rodent Reticulocyte enriched Erythrocyte Population (REP) compared to wild type Erythrocyte Population (wtEP). See S1 Table for metabolite names corresponding to numbers. **Fig B.** A. Schematic representation of gene deletion strategy. B. Gel electrophoresis of indicated PCR products to confirm integration of selection cassette, disruption of genes and clonality of mutant parasites (i) *pepc* (PBANKA_101790) (ii) *mdh* (PBANKA_111770) (iii) *oprt* (PBANKA_111240) (iv) *ompdc* (PBANKA_050740). **Fig C.** A. Competition growth assay using FACS analysis. Equal number of parasites (10^6^) of wt population expressing RFP under constitutive promoter eef1a (RMgm-86) and mutant population made in a parent line expressing GFP under the same promoter (RMgm-7) were mixed and injected into a mouse on day 0 and peripheral blood from the infected mouse was monitored using FACS analyses for the proportion of RFP positive (wt) and GFP positive (mutant) parasites over the next 12 days. Infected cells were gated on forward-side scatter followed by Hoechst 33258 staining and then on GFP and RFP staining as shown in the representative FACS plots. Left panel on day 0 shows wt and mutant populations are in similar proportions and right panel shows that over time (by days 6–12), wt population overgrows a slow growing mutant. Infected blood was passaged into a new mouse when multiple infected cells started to appear to allow for optimal growth. B. Time taken for asexual parasites to grow to mature schizont stage. Coloured lines indicate non-linear fit of percentage of mature schizonts observed in *in vitro* synchronous cultures of wt and mutant *P*. *berghei* parasites 22 hours post invasion. Data representative of n = 3 independent biological replicates. C. Number of merozoites per schizont grown in *in vitro* cultures as counted in giemsa stained smears. The error is given as the SD of n ≥ 40 schizonts. Data representative of 3 independent biological replicates. P-values: ***p<0.0005, unpaired two tailed t-test. D. FACS plots showing DNA replication in male gametocytes observed by FACS analysis at the start of and 12 mins post activation. DNA content was determined in Hoechst-33258-stained purified gametocytes and flouroscence intensity is displayed on x-axis and cell counts on y- axis. Before activation (0 min) males and females are shown in a gate with the same low DNA content (M+F). At 12 mins, prior to the formation of free male gametes, the DNA content of males increases as shown in gate (M). E. Left panel: Intensity of Giemsa staining in wt and mutant *P*. *berghei* infected reticulocytes. Intensity of the stain was found to be maximum in youngest reticulocytes. Smears were stained with 12% Giemsa stain for 10 mins after fixing and air drying with methanol. Image data was processed in ImageJ. Gray values were calculated in minimum 100 pixels across the whole host cell (parasites inside the cells were excluded) and staining intensities (Gray value^-1^ normalised to mature erythrocytes) were plotted for n = 20 infected cells. P-values: **p<0.005, ***p<0.0005, paired two tailed t-test. Right panel: representative pictures showing *P*. *berghei* infected erythrocytes in smears stained with Giemsa where younger reticulocytes are stained darker. **Fig D.** A. Mosquito mid guts showing mature oocysts at day 14 post infection in wt, *pepc* ^-^ and *mdh* ^-^ *P*.*berghei* infected mosquitoes. B. Mosquito salivary glands showing sporozoites at day 21 post infection in wt, *pepc* ^-^ and *mdh* ^-^ *P*.*berghei* infected mosquitoes. C. Mosquito mid guts showing mature oocysts at day 14 post infection in wt, *oprt* ^-^ and *ompdc* ^-^ *P*.*berghei* infected mosquitoes. D. Mosquito salivary glands showing sporozoites at day 21 post infection in wt, *oprt* ^-^ and *ompdc* ^-^ *P*.*berghei* infected mosquitoes. **Fig E.** Mosquito infectivity as observed by counting mature fluorescent oocysts of *in vivo* crossed Pb270 x *oprt-* and Pb270 x *ompdc-* mutant parasites compared to wt and self-fertilised Pb270 on day 14 post infected blood feed in mosquito mid guts. n = 20 mosquitoes cumulative of two independent biological replicates. ***p<0.0005, unpaired two tailed t-test. **Fig F.** A. Schematic representation of glutathione synthesis pathway in *Plasmodium*. *ɣ-GCS* (ɣ-glutamylcysteine synthetase), *GS* (glutathionesSynthetase), *GR* (glutathione reductase) ɣ-GluCys (ɣ-L-glutamyl-L-cysteine), GSSG (glutathione disulphide). B. Fold change of relative levels (peak intensities) of metabolites of glutathione biosynthesis in rodent REP compared to wtEP. Dotted line indicates no change and error bars indicate R.S.D. (Relative Standard Deviation) of peak intensities from reticulocyte samples multiplied to the fold change values from n = 3 independent biological replicates. It is expected that under these metabolomics extraction conditions the oxidised forms of cystine and glutathione disulphide likely represent the sum of both oxidised and reduced forms of cysteine and glutathione. C. *P*. *berghei* inhibition experiment with buthionine sulphoximine (BSO) *in vitro*. Error bars indicate S.D. from n = 2 biological replicates. **Fig G.** Illustration of ICM and Pyrimidine metabolism genes’ essentiality throughout *P*. *berghei* life cycle.
(PDF)

S1 Table Metabolites represented in Fig 1B showing fold change in abundance in uninfected Reticulocyte enriched Erythrocyte Population (REP)compared to wild type Erythrocyte Population (wtEP) from rats. Metabolites are listed in order of decreasing abundance. Metabolites identified with authentic standards are highlighted bold, others are considered putative identifications.
(DOCX)

S2 Table List of primers used for generation of gene knockouts and confirmation. (DOCX)

S3 Table List of genes annotated with metabolic pathway information in *P*. *falciparum* on PlasmoDB and their *P*. *vivax* and *P*. *berghei* orthologues. (XLSX)

S1 Supplementary Materials and Methods Details of materials and methods used for metabolomics sample preparation, data acquisition and analysis, generation of gene deletion mutants in *P*. *berghei* and phenotypic analysis. (DOCX)
