# Supplementary material for: Host Reticulocytes Provide Metabolic Reservoirs That Can Be Exploited by Malaria Parasites
Source: PLoS Pathog. 2015 Jun 4;11(6):e1004882. doi: 10.1371/journal.ppat.1004882 (PMC4456406; doi:10.1371/journal.ppat.1004882)
Supplement: S1 Text — A. Characterisation of the enriched reticulocyte population induced by Phenylhydrazine-HCl (PHZ) by FACS analysis on day 5 post PHZ administration. Top panel shows Ter119-FITC staining in RBCs which stains all erythroid cells. Bottom panels show CD71-APC staining in RBCs which stains only reticulocytes (~35%) and the population of CD71-high reticulocytes is almost 90% indicating that the majority of reticulocytes are very young. B. Volcano plot showing the distribution of abundance of all ~4560 peaks detected across both LC-MS and GC-MS platforms in Reticulocyte enriched Erythrocyte Population (REP) as compared to wild type Erythrocyte Population (wtEP) in rodent blood. All significant changes are represented above the broken horizontal line. Coloured dots indicate peaks which are: Blue- significantly up-regulated, Red- significantly down-regulated, Yellow- significant but little change, Brown- non-significant. n = 3 independent biological replicates (with four internal technical replicates each). Significance tested by Welch’s T-test (< 0.05). C. Fold change of metabolite abundance in rodent Reticulocyte enriched Erythrocyte Population (REP) compared to wild type Erythrocyte Population (wtEP). See S1 Table for metabolite names corresponding to numbers. Fig B. A. Schematic representation of gene deletion strategy. B. Gel electrophoresis of indicated PCR products to confirm integration of selection cassette, disruption of genes and clonality of mutant parasites (i) pepc (PBANKA_101790) (ii) mdh (PBANKA_111770) (iii) oprt (PBANKA_111240) (iv) ompdc (PBANKA_050740). Fig C. A. Competition growth assay using FACS analysis. Equal number of parasites (106) of wt population expressing RFP under constitutive promoter eef1a (RMgm-86) and mutant population made in a parent line expressing GFP under the same promoter (RMgm-7) were mixed and injected into a mouse on day 0 and peripheral blood from the infected mouse was monitored using FACS analyses for the proportion of RFP pos [file ppat.1004882.s001.pdf]

Fig. A

A

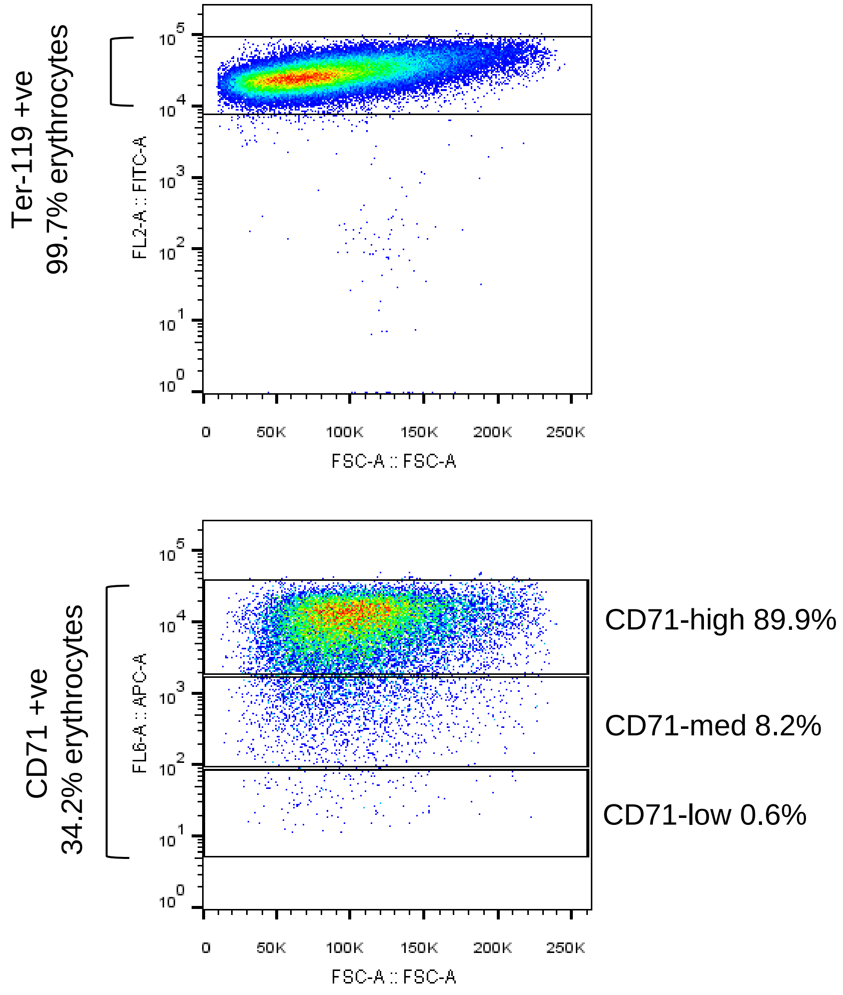
Ter-119 +ve
99.7% erythrocytes
FL2-A :: FITC-A
FSC-A :: FSC-A
0
50K
100K
150K
200K
250K
CD71 +ve
34.2% erythrocytes
FL6-A :: APC-A
CD71-high 89.9%
CD71-med 8.2%
CD71-low 0.6%
FSC-A :: FSC-A

B

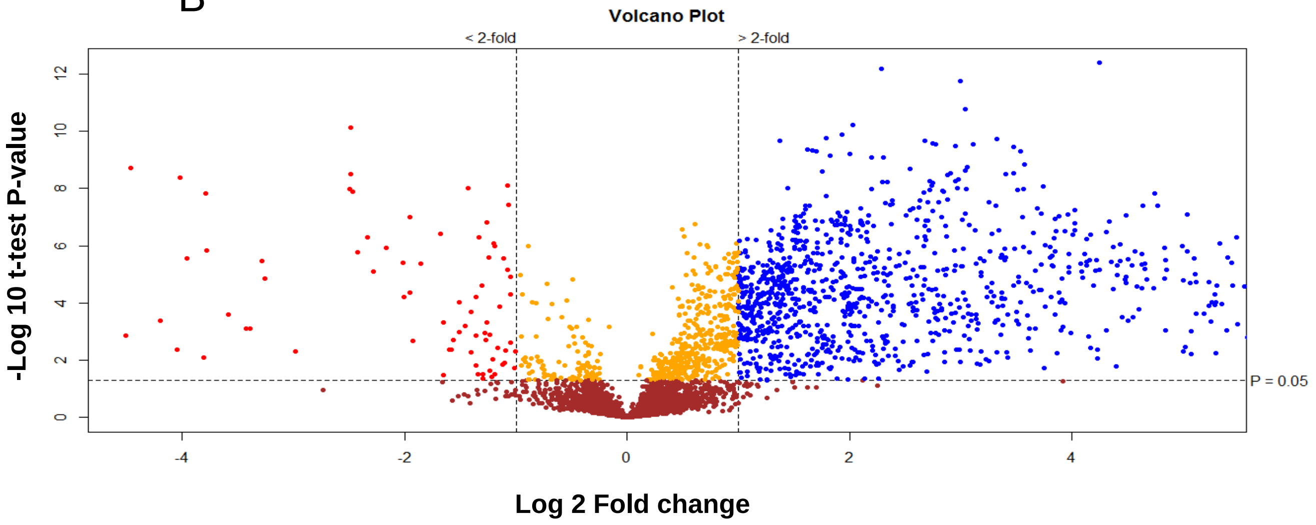
Volcano Plot
< 2-fold
> 2-fold
P = 0.05
-Log 10 t-test P-value
0
2
4
6
8
10
12
-4
-2
0
2
4
Log 2 Fold change

S1 Text

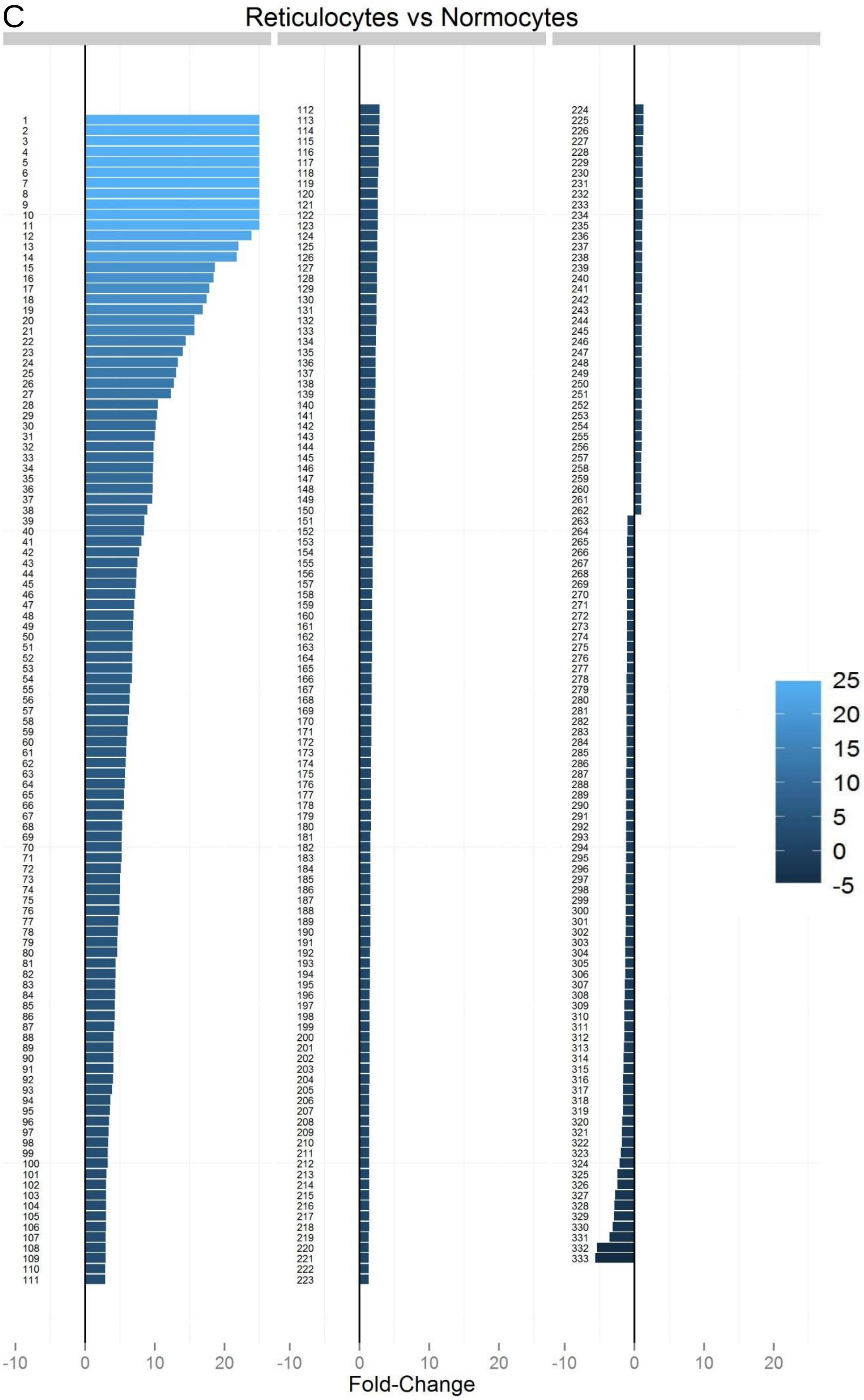

# Fig. B

## A

### (i) *pepc*[-] (PBANKA_101790)

| Primers | Product | Size |
|---|---|---|
| GU2057+GU2061 | 5' integration | 1kb |
| GU0204+GU2058 | 3' integration | 1.1kb |
| GU2059+GU2060 | ORF | 1kb |

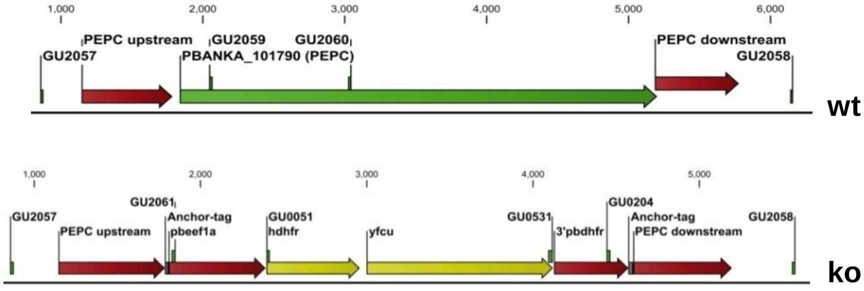


### (ii) *mdh*[-] (PBANKA_111770)

| Primers | Product | Size |
|---|---|---|
| GU2278+GU2061 | 5' integration | 1.3kb |
| GU0204+GU2279 | 3' integration | 0.75kb |
| GU2280+GU2281 | ORF | 0.87kb |

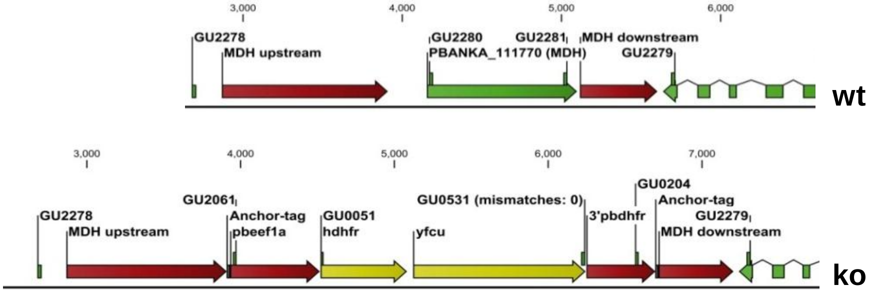


### (iii) *oprt*[-] (PBANKA_111240)

| Primers | Product | Size |
|---|---|---|
| GU2286+GU2061 | 5' integration | 0.6kb |
| GU0204+GU2610 | 3' integration | 1.0kb |
| GU2288+GU2289 | ORF | 0.7kb |

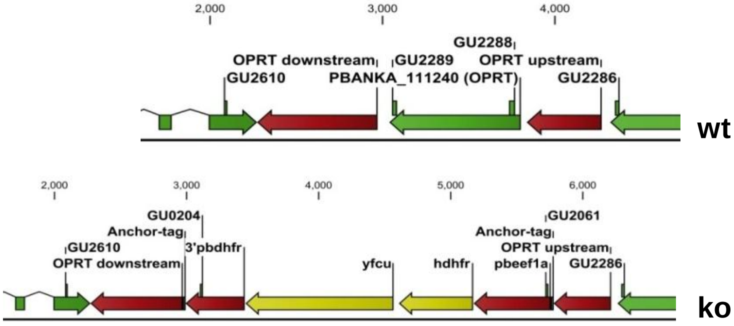


### (iv) *ompdc*[-] (PBANKA_050740)

| Primers | Product | Size |
|---|---|---|
| GU2282+GU2061 | 5' integration | 1.2kb |
| GU0204+GU2283 | 3' integration | 1.4kb |
| GU2284+GU2285 | ORF | 0.9kb |

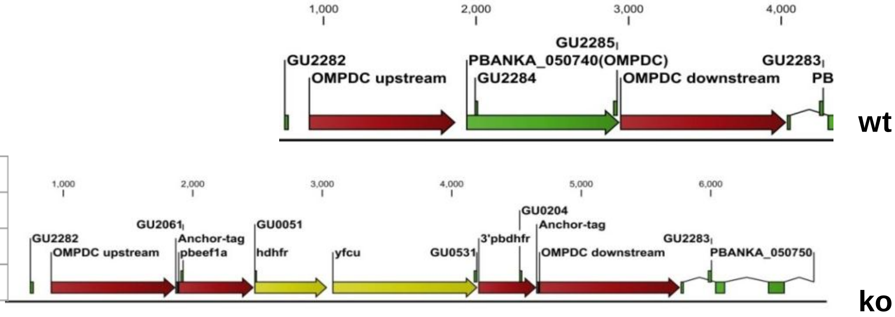

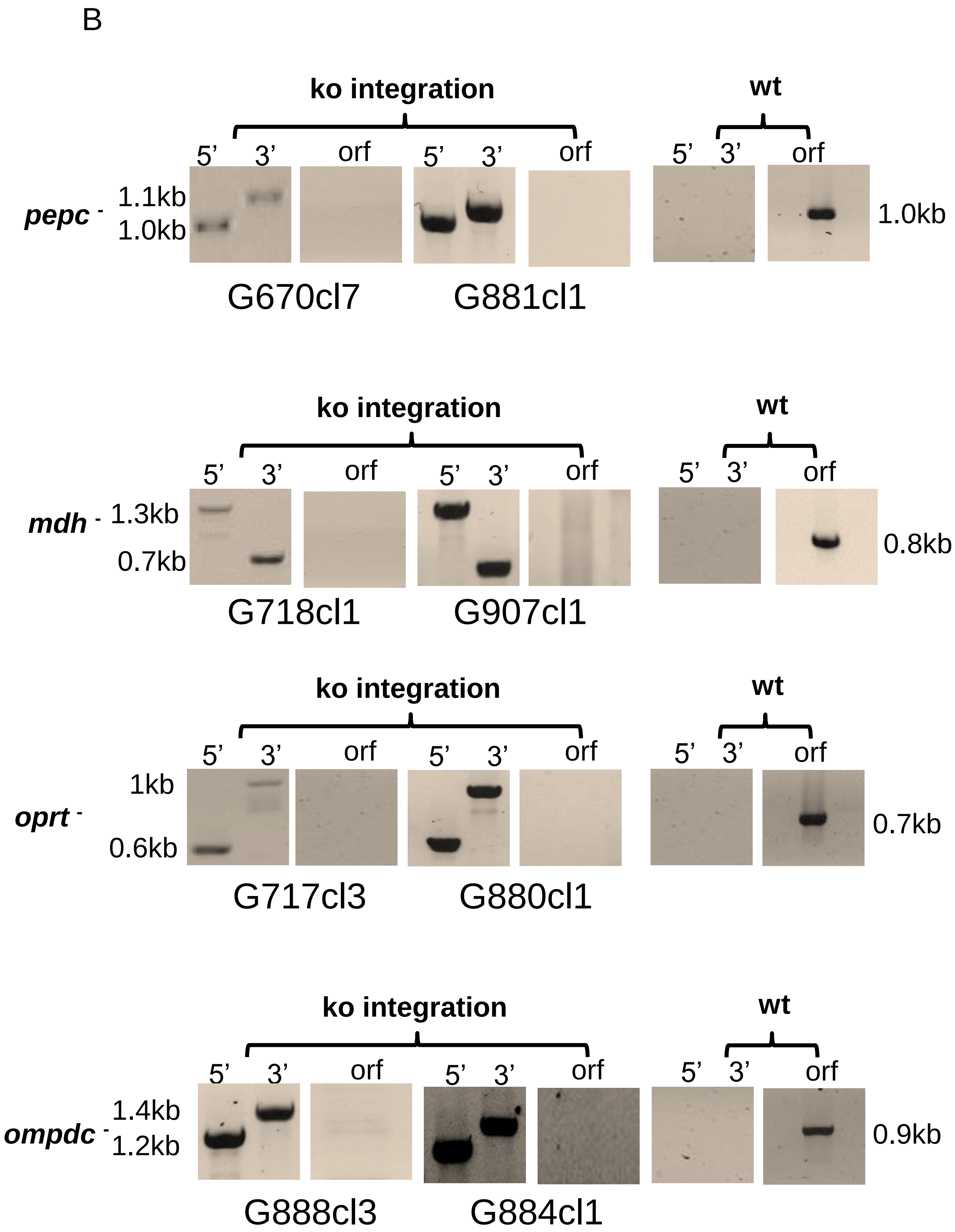
B
ko integration
wt
5' 3' orf 5' 3' orf
5' 3' orf
pepc -
1.1kb
1.0kb
1.0kb
G670cl7
G881cl1
mdh -
1.3kb
0.7kb
0.8kb
G718cl1
G907cl1
oprt -
1kb
0.6kb
0.7kb
G717cl3
G880cl1
ompdc -
1.4kb
1.2kb
0.9kb
G888cl3
G884cl1

Fig. C

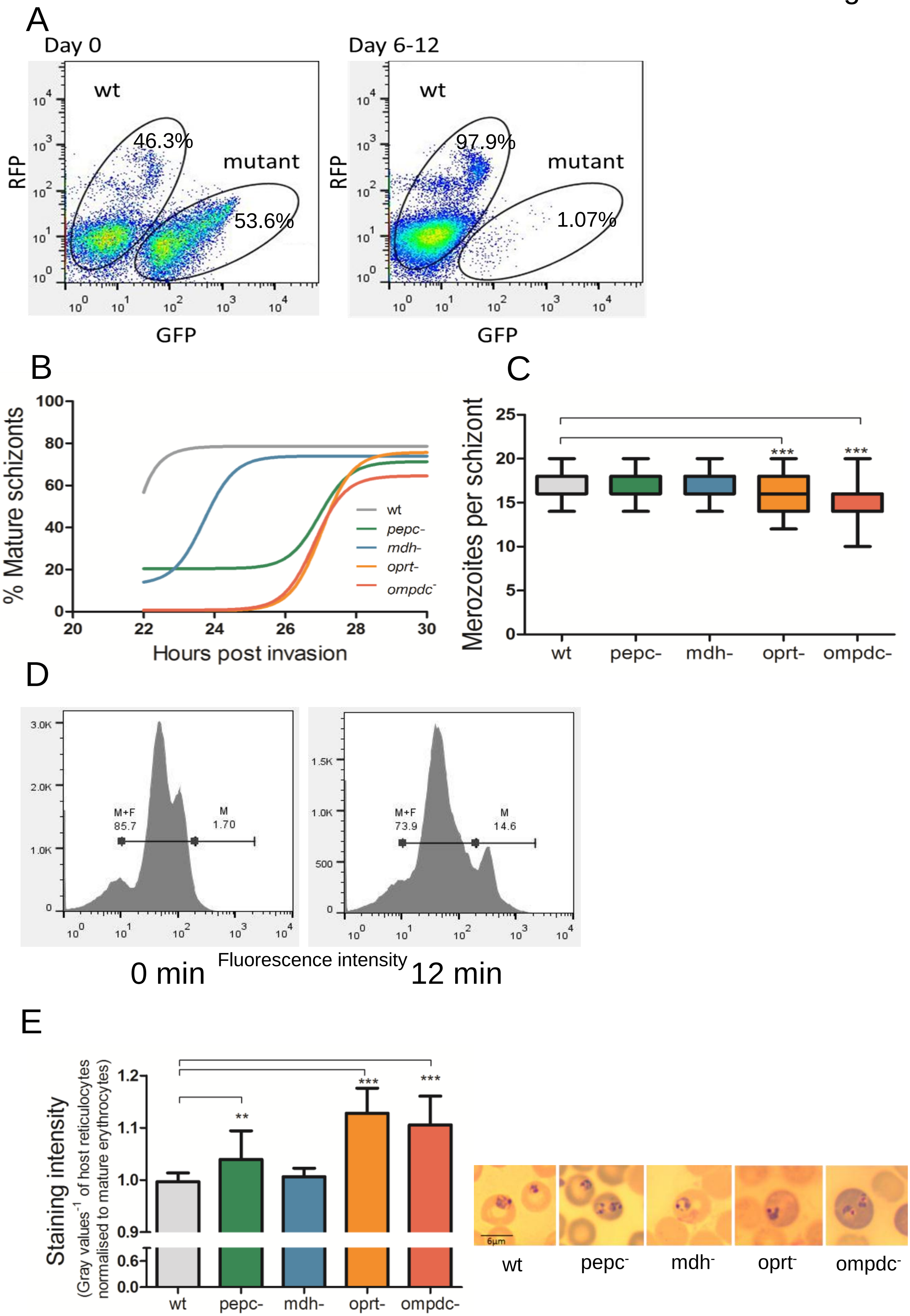
A
Day 0
Day 6-12
wt
mutant
46.3%
53.6%
97.9%
1.07%
RFP
GFP
B
% Mature schizonts
Hours post invasion
wt
pepc-
mdh-
oprt-
ompdc-
C
Merozoites per schizont
***
***
wt
pepc-
mdh-
oprt-
ompdc-
D
M+F
85.7
M
1.70
M+F
73.9
M
14.6
0 min
Fluorescence intensity
12 min
E
Staining intensity
(Gray values-1 of host reticulocytes normalised to mature erythrocytes)
**
***
***
wt
pepc-
mdh-
oprt-
ompdc-
6µm
wt
pepc-
mdh-
oprt-
ompdc-

Fig. D

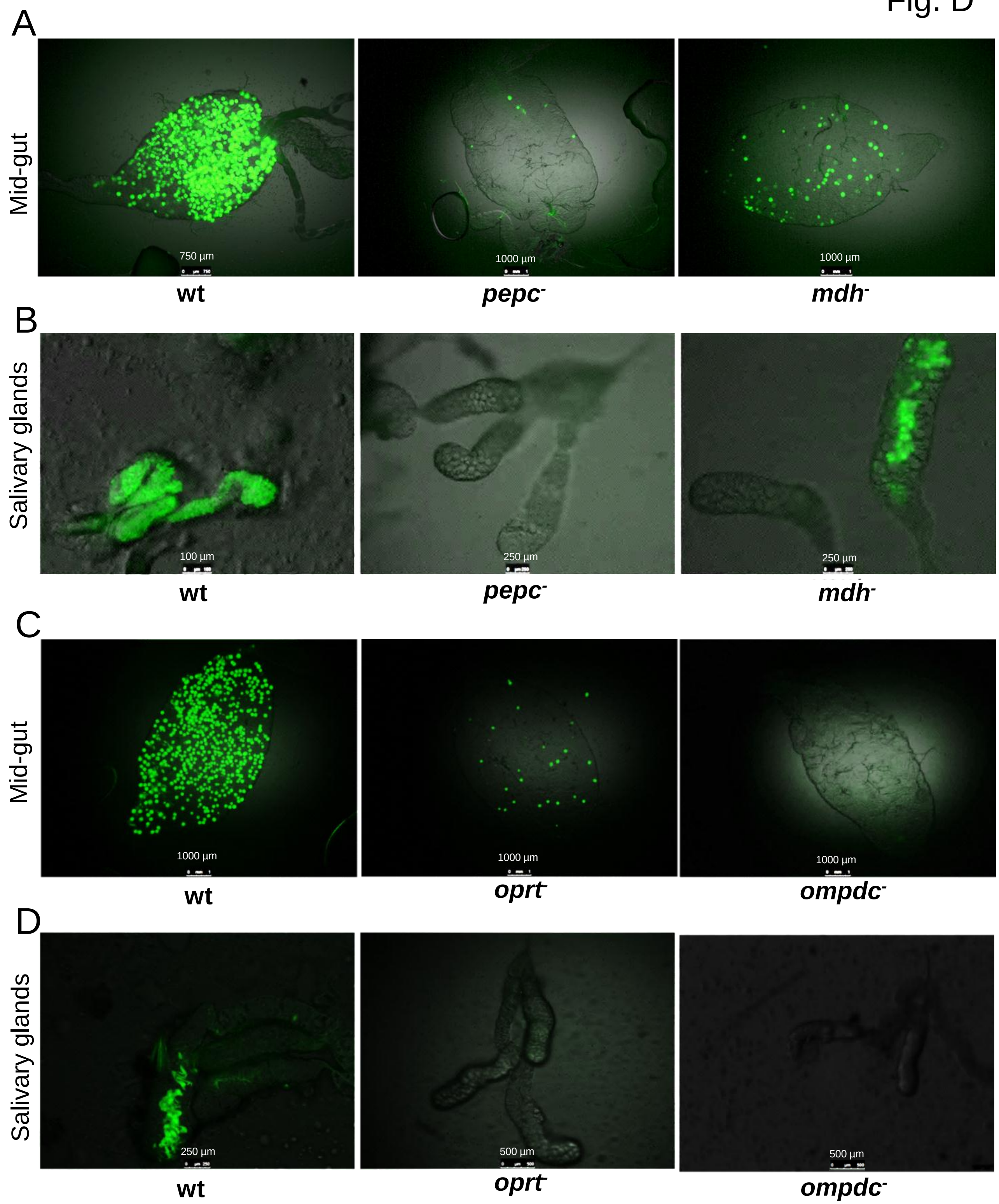
A
Mid-gut
750 µm
1000 µm
1000 µm
wt
pepc-
mdh-
B
Salivary glands
100 µm
250 µm
250 µm
wt
pepc-
mdh-
C
Mid-gut
1000 µm
1000 µm
1000 µm
wt
oprt-
ompdc-
D
Salivary glands
250 µm
500 µm
500 µm
wt
oprt-
ompdc-

Fig. E

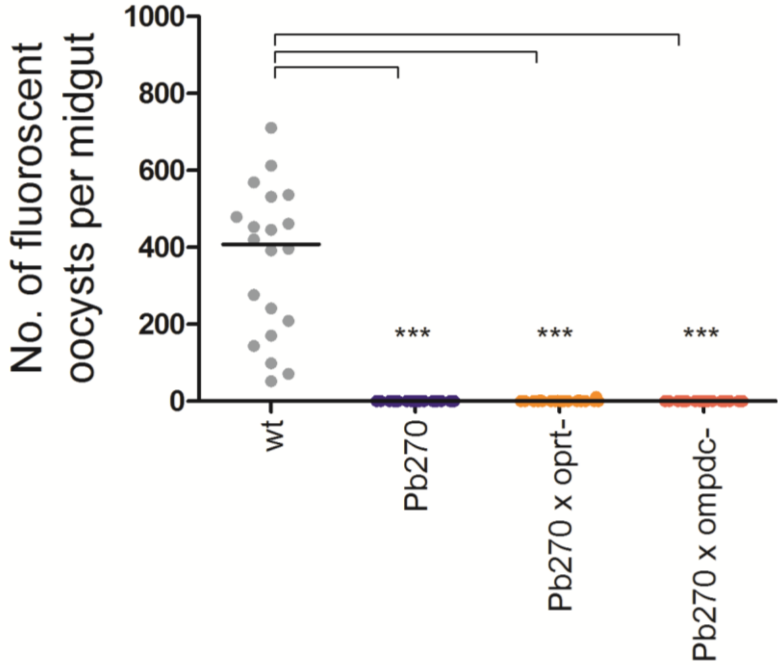
No. of fluoroscent oocysts per midgut
1000
800
600
400
200
0
***
***
***
wt
Pb270
Pb270 x oprt-
Pb270 x ompdc-

Fig. F

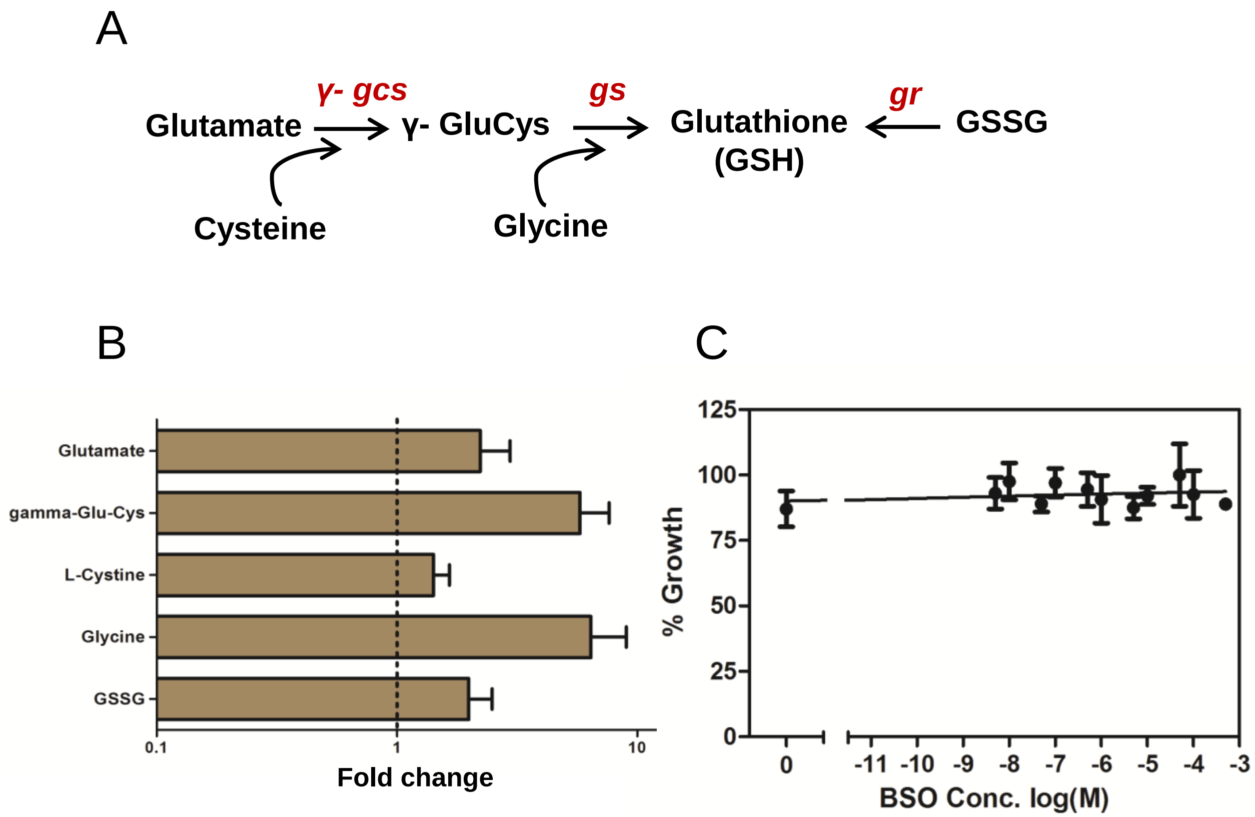
A
γ- gcs
gs
gr
Glutamate
γ- GluCys
Glutathione (GSH)
GSSG
Cysteine
Glycine
B
Glutamate
gamma-Glu-Cys
L-Cystine
Glycine
GSSG
0.1
1
10
Fold change
C
% Growth
125
100
75
50
25
0
0
-11 -10 -9 -8 -7 -6 -5 -4 -3
BSO Conc. log(M)

Fig. G

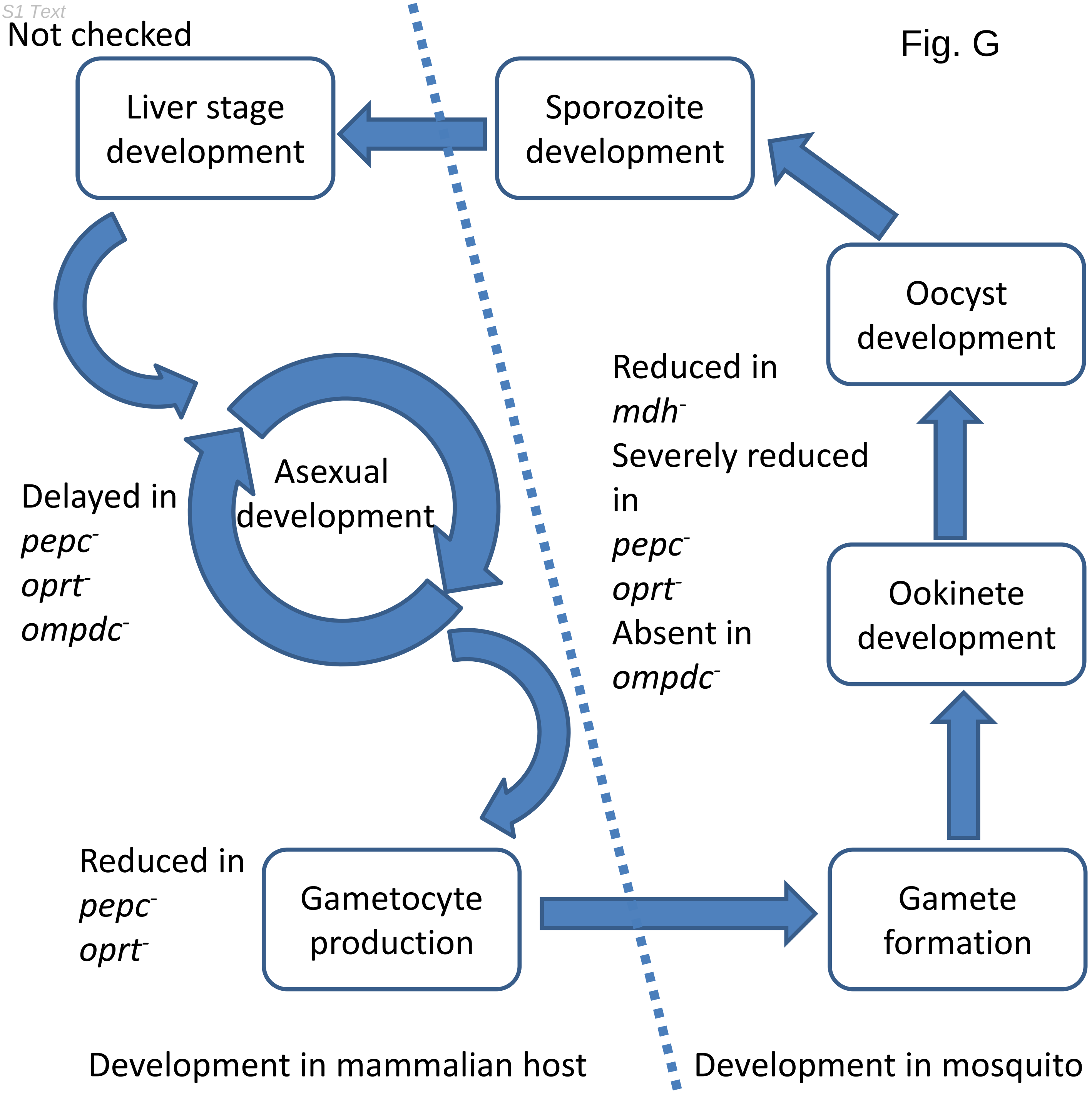
Not checked
Liver stage development
Sporozoite development
Oocyst development
Reduced in *mdh*$^-$
Severely reduced in *pepc*$^-$ *oprt*$^-$
Absent in *ompdc*$^-$
Asexual development
Delayed in *pepc*$^-$ *oprt*$^-$ *ompdc*$^-$
Ookinete development
Reduced in *pepc*$^-$ *oprt*$^-$
Gametocyte production
Gamete formation
Development in mammalian host
Development in mosquito
