# Supplementary material for: Host Reticulocytes Provide Metabolic Reservoirs That Can Be Exploited by Malaria Parasites
Source: PLoS Pathog. 2015 Jun 4;11(6):e1004882. doi: 10.1371/journal.ppat.1004882 (PMC4456406; doi:10.1371/journal.ppat.1004882)
Supplement: S1 Table — Metabolites are listed in order of decreasing abundance. Metabolites identified with authentic standards are highlighted bold, others are considered putative identifications. (DOCX) [file ppat.1004882.s002.docx]

# Table S1

List of metabolites represented in Figure 1B.

| **No.** | **Putative Metabolite** | **Fold change** | **Fold change** (corrected for presence of 65% mature erythrocytes) | **P-value** | **Platform** |
| --- | --- | --- | --- | --- | --- |
| 1 | Citric acid | >25 fold | >25 fold | 9.65E-07 | GC-MS |
| 2 | UMP | >25 fold | >25 fold | 1.03E-02 | GC-MS |
| 3 | Nonanoylcarnitine | >25 fold | >25 fold | 8.40E-06 | LC-MS |
| 4 | S-Methyl-L-methionine | >25 fold | >25 fold | 1.01E-05 | LC-MS |
| 5 | Ala-Val-Pro-Ser | >25 fold | >25 fold | 1.64E-06 | LC-MS |
| 6 | Dihydrobiopterin | >25 fold | >25 fold | 9.59E-05 | LC-MS |
| 7 | N-Acetyl-aspartyl-glutamate | >25 fold | >25 fold | 3.80E-11 | LC-MS |
| 8 | 2-Amino-4-hydroxy-6-hydroxymethyl-7,8-dihydropteridine | >25 fold | >25 fold | 3.98E-06 | LC-MS |
| 9 | Aspartyl-L-proline | >25 fold | >25 fold | 4.06E-05 | LC-MS |
| 10 | sn-glycero-3-Phospho-1-inositol | >25 fold | >25 fold | 2.48E-06 | LC-MS |
| 11 | **L-Aspartate** | >25 fold | >25 fold | 1.08E-05 | LC-MS |
| 12 | Dodecanoylcarnitine | 23.92 | >25 fold | 6.91E-06 | LC-MS |
| 13 | CDP-choline | 22.04 | >25 fold | 3.28E-04 | LC-MS |
| 14 | UDP-N-acetyl-D-glucosamine | 21.79 | >25 fold | 2.95E-08 | LC-MS |
| 15 | O-Propanoylcarnitine | 18.66 | >25 fold | 3.14E-06 | LC-MS |
| 16 | Glycerophosphoglycerol | 18.45 | >25 fold | 9.04E-05 | LC-MS |
| 17 | **L-Carnitine** | 17.81 | >25 fold | 3.18E-06 | LC-MS |
| 18 | L-Octanoylcarnitine | 17.47 | >25 fold | 5.88E-07 | LC-MS |
| 19 | Glu-Asp | 16.92 | >25 fold | 9.51E-07 | LC-MS |
| 20 | N6-Acetyl-N6-hydroxy-L-lysine | 15.71 | >25 fold | 2.71E-04 | LC-MS |
| 21 | **Orotate** | 15.68 | >25 fold | 1.00E-07 | LC-MS |
| 22 | dCMP-ethanolamine | 14.45 | >25 fold | 5.94E-06 | LC-MS |
| 23 | Tetradecanoylcarnitine | 13.99 | >25 fold | 4.84E-06 | LC-MS |
| 24 | Sedoheptulose | 13.30 | >25 fold | 1.02E-06 | LC-MS |
| 25 | Gamma Glutamyl-glutamic acid | 13.10 | >25 fold | 1.13E-04 | LC-MS |
| 26 | Creatinine phosphate | 12.79 | >25 fold | 3.60E-03 | LC-MS |
| 27 | Phosphocreatine | 12.32 | 24.64 | 4.50E-03 | LC-MS |
| 28 | [GP (16:0)] 1-hexadecanoyl-2-sn-glycero-3-phosphate | 10.43 | 20.86 | 1.81E-06 | LC-MS |
| 29 | Glu-Pro | 10.31 | 20.62 | 7.35E-05 | LC-MS |
| 30 | L-Tyrosine methyl ester | 10.15 | 20.3 | 2.82E-07 | LC-MS |
| 31 | CDP-ethanolamine | 10.03 | 20.06 | 3.21E-05 | LC-MS |
| 32 | Ala-Asp-Asp | 9.86 | 19.72 | 3.46E-06 | LC-MS |
| 33 | dTTP | 9.84 | 19.68 | 5.15E-07 | LC-MS |
| 34 | **CMP** | 9.75 | 19.5 | 7.49E-06 | LC-MS |
| 35 | Prenyl-L-cysteine | 9.73 | 19.46 | 5.95E-05 | LC-MS |
| 36 | N2-Succinyl-L-ornithine | 9.68 | 19.36 | 3.84E-07 | LC-MS |
| 37 | N-(3S-hydroxydecanoyl)-L-serine | 9.63 | 19.26 | 4.14E-05 | LC-MS |
| 38 | Met-Thr-Asp | 8.97 | 17.94 | 1.15E-05 | LC-MS |
| 39 | **Malate** | 8.55 | 17.1 | 2.82E-05 | LC-MS |
| 40 | Ala-Ser-Tyr | 8.46 | 16.92 | 5.50E-04 | LC-MS |
| 41 | 2,3,4,5-Tetrahydrodipicolinate | 8.05 | 16.1 | 6.81E-05 | LC-MS |
| 42 | **IMP** | 7.75 | 15.5 | 5.83E-07 | LC-MS |
| 43 | Ala-Cys | 7.53 | 15.06 | 5.07E-06 | LC-MS |
| 44 | **Xanthine** | 7.36 | 14.72 | 1.10E-08 | LC-MS |
| 45 | **Ribulose-5-phosphate** | 7.33 | 14.66 | 7.69E-03 | GC-MS |
| 46 | Acetylcholine | 7.19 | 14.38 | 6.92E-06 | LC-MS |
| 47 | **Choline phosphate** | 7.10 | 14.2 | 1.59E-04 | LC-MS |
| 48 | Glu-Gly | 6.92 | 13.84 | 7.27E-06 | LC-MS |
| 49 | **Fumarate** | 6.90 | 13.8 | 1.03E-04 | LC-MS |
| 50 | O-Butanoylcarnitine | 6.84 | 13.68 | 2.96E-10 | LC-MS |
| 51 | N-Acetyl-D-mannosamine | 6.80 | 13.6 | 9.13E-07 | LC-MS |
| 52 | N-Carbamoyl-L-aspartate | 6.78 | 13.56 | 1.94E-05 | LC-MS |
| 53 | Dihydroorotate | 6.76 | 13.52 | 1.82E-06 | LC-MS |
| 54 | Spermidine | 6.71 | 13.42 | 2.74E-10 | LC-MS |
| 55 | allylcysteine | 6.42 | 12.84 | 6.51E-07 | LC-MS |
| 56 | **Glycine** | 6.41 | 12.82 | 1.50E-05 | LC-MS |
| 57 | Thiomorpholine 3-carboxylate | 6.35 | 12.7 | 5.69E-06 | LC-MS |
| 58 | 1-methylguanosine | 6.11 | 12.22 | 1.89E-08 | LC-MS |
| 59 | Gly-Pro | 6.06 | 12.12 | 1.32E-07 | LC-MS |
| 60 | Glu-Leu | 5.95 | 11.9 | 1.50E-04 | LC-MS |
| 61 | Leu-Thr | 5.86 | 11.72 | 2.49E-05 | LC-MS |
| 62 | Thr-Ala | 5.85 | 11.7 | 1.05E-04 | LC-MS |
| 63 | gamma-L-Glutamyl-L-cysteine | 5.77 | 11.54 | 1.66E-06 | LC-MS |
| 64 | 1-Methyladenosine | 5.69 | 11.38 | 9.08E-08 | LC-MS |
| 65 | 3-Hydroxy-N6,N6,N6-trimethyl-L-lysine | 5.60 | 11.2 | 1.67E-02 | LC-MS |
| 66 | O-hexanoyl-R-carnitine | 5.54 | 11.08 | 5.41E-04 | LC-MS |
| 67 | O-decanoyl-R-carnitine | 5.35 | 10.7 | 5.33E-06 | LC-MS |
| 68 | Malonylcarnitine | 5.34 | 10.68 | 3.57E-04 | LC-MS |
| 69 | **D-Ribose 5-phosphate** | 5.29 | 10.58 | 3.61E-11 | LC-MS |
| 70 | glucosamine-1,6-diphosphate | 5.28 | 10.56 | 2.45E-11 | LC-MS |
| 71 | N2-Acetyl-L-aminoadipate | 5.24 | 10.48 | 1.24E-04 | LC-MS |
| 72 | Leu-Pro | 5.13 | 10.26 | 2.77E-06 | LC-MS |
| 73 | 2-Hydroxyadenine | 5.02 | 10.04 | 1.52E-06 | LC-MS |
| 74 | NG,NG-Dimethyl-L-arginine | 4.99 | 9.98 | 2.55E-03 | LC-MS |
| 75 | Fructoselysine 6-phosphate | 4.95 | 9.9 | 2.90E-10 | LC-MS |
| 76 | **D-Gluconic acid** | 4.95 | 9.9 | 1.05E-04 | LC-MS |
| 77 | D-Xylulose | 4.77 | 9.54 | 1.06E-06 | LC-MS |
| 78 | S-Methyl glutathione | 4.72 | 9.44 | 1.54E-05 | LC-MS |
| 79 | **sn-Glycerol 3-phosphate** | 4.67 | 9.34 | 4.01E-11 | LC-MS |
| 80 | Pseudouridine | 4.64 | 9.28 | 3.97E-07 | LC-MS |
| 81 | **Succinate** | 4.41 | 8.82 | 2.10E-03 | LC-MS |
| 82 | **Cytidine** | 4.38 | 8.76 | 1.22E-03 | LC-MS |
| 83 | Monomethyl-arginine | 4.32 | 8.64 | 5.82E-03 | LC-MS |
| 84 | Gamma-Aminobutyryl-lysine | 4.30 | 8.6 | 1.56E-07 | LC-MS |
| 85 | 5-Methylcytidine | 4.27 | 8.54 | 4.33E-09 | LC-MS |
| 86 | Glu-Val | 4.25 | 8.5 | 7.05E-05 | LC-MS |
| 87 | Ala-Pro | 4.22 | 8.44 | 5.39E-08 | LC-MS |
| 88 | Pro-Pro | 4.10 | 8.2 | 2.73E-06 | LC-MS |
| 89 | [PC (16:0)] 1-hexadecanoyl-sn-glycero-3-phosphocholine | 4.10 | 8.2 | 1.07E-04 | LC-MS |
| 90 | -Hydroxy-eicosatetraenoic acid | 4.10 | 8.2 | 2.54E-03 | LC-MS |
| 91 | Xanthosine | 4.05 | 8.1 | 8.44E-09 | LC-MS |
| 92 | N-Acetyl-D-glucosamine 6-sulfate | 4.00 | 8 | 1.12E-07 | LC-MS |
| 93 | N-Acetyl-L-aspartate | 3.91 | 7.82 | 4.80E-07 | LC-MS |
| 94 | **Uridine** | 3.63 | 7.26 | 8.83E-07 | LC-MS |
| 95 | **3',5'-Cyclic AMP** | 3.59 | 7.18 | 2.75E-05 | LC-MS |
| 96 | N1-Acetylspermidine | 3.44 | 6.88 | 1.54E-05 | LC-MS |
| 97 | Glu-Ser | 3.37 | 6.74 | 1.74E-05 | LC-MS |
| 98 | **N-Acetylneuraminate** | 3.33 | 6.66 | 4.47E-05 | LC-MS |
| 99 | N-Acetyl-L-glutamate 5-semialdehyde | 3.28 | 6.56 | 5.02E-06 | LC-MS |
| 100 | Val-Val | 3.25 | 6.5 | 7.95E-07 | LC-MS |
| 101 | Asp-Asp | 3.07 | 6.14 | 4.30E-10 | LC-MS |
| 102 | Erythrulose 1-phosphate | 3.03 | 6.06 | 3.51E-05 | LC-MS |
| 103 | Glu-Cys-Gln-Gln | 3.01 | 6.02 | 9.51E-06 | LC-MS |
| 104 | **Choline** | 3.01 | 6.02 | 4.04E-05 | LC-MS |
| 105 | N-Acetylserotonin | 3.00 | 6 | 2.80E-05 | LC-MS |
| 106 | Ala-Leu-Lys-Pro | 2.99 | 5.98 | 1.31E-02 | LC-MS |
| 107 | (1-Ribosylimidazole)-4-acetate | 2.97 | 5.94 | 4.71E-08 | LC-MS |
| 108 | O-Acetyl-L-homoserine | 2.94 | 5.88 | 4.52E-04 | LC-MS |
| 109 | 1-Methylnicotinamide | 2.93 | 5.86 | 4.96E-04 | LC-MS |
| 110 | D-myo-Inositol 1,2-cyclic phosphate | 2.92 | 5.84 | 7.41E-05 | LC-MS |
| 111 | 2-Carboxy-D-arabinitol 1-phosphate | 2.91 | 5.82 | 2.91E-06 | LC-MS |
| 112 | Glu-Thr | 2.88 | 5.76 | 7.89E-05 | LC-MS |
| 113 | **D-Erythrose 4-phosphate** | 2.86 | 5.72 | 5.47E-10 | LC-MS |
| 114 | Ala-Asp-Cys | 2.82 | 5.64 | 3.04E-07 | LC-MS |
| 115 | N3-(4-methoxyfumaroyl)-L-2,3-diaminopropanoate | 2.78 | 5.56 | 2.32E-06 | LC-MS |
| 116 | Ethanolamine phosphate | 2.74 | 5.48 | 2.44E-03 | LC-MS |
| 117 | Taurine | 2.71 | 5.42 | 1.13E-03 | LC-MS |
| 118 | Taurocyamine | 2.70 | 5.4 | 2.20E-04 | LC-MS |
| 119 | N-(L-Arginino)succinate | 2.64 | 5.28 | 5.09E-05 | LC-MS |
| 120 | **L-Arginine** | 2.62 | 5.24 | 5.82E-06 | LC-MS |
| 121 | **D-Glucose 6-phosphate** | 2.61 | 5.22 | 9.22E-05 | LC-MS |
| 122 | [SP] 3-dehydrosphinganine | 2.60 | 5.2 | 8.90E-05 | LC-MS |
| 123 | [SP] Sphing-4-enine-1-phosphate | 2.59 | 5.18 | 2.21E-10 | LC-MS |
| 124 | CMP-N-acetylneuraminate | 2.53 | 5.06 | 1.72E-05 | LC-MS |
| 125 | N2-(D-1-Carboxyethyl)-L-lysine | 2.53 | 5.06 | 1.59E-03 | LC-MS |
| 126 | Acetyl phosphate | 2.51 | 5.02 | 1.02E-05 | LC-MS |
| 127 | Hexose-phosphate | 2.47 | 4.94 | 1.08E-04 | LC-MS |
| 128 | Leucyl-leucine | 2.46 | 4.92 | 4.24E-05 | LC-MS |
| 129 | N-(octanoyl)-L-homoserine | 2.46 | 4.92 | 2.88E-07 | LC-MS |
| 130 | **Putrescine** | 2.44 | 4.88 | 1.47E-06 | LC-MS |
| 131 | **AMP** | 2.44 | 4.88 | 6.68E-03 | LC-MS |
| 132 | 3-sulfopropanoate | 2.42 | 4.84 | 8.40E-04 | LC-MS |
| 133 | Leu-Val | 2.39 | 4.78 | 4.89E-06 | LC-MS |
| 134 | D-Methionine | 2.35 | 4.7 | 9.33E-04 | LC-MS |
| 135 | Ala-ala | 2.31 | 4.62 | 3.03E-02 | LC-MS |
| 136 | **Hypoxanthine** | 2.29 | 4.58 | 4.84E-02 | LC-MS |
| 137 | N5-Ethyl-L-glutamine | 2.29 | 4.58 | 1.86E-02 | LC-MS |
| 138 | N-Acetylglutamine | 2.28 | 4.56 | 4.27E-05 | LC-MS |
| 139 | **L-Glutamate** | 2.22 | 4.44 | 1.01E-04 | LC-MS |
| 140 | **L-Ornithine** | 2.22 | 4.44 | 2.29E-02 | LC-MS |
| 141 | Phe-Pro | 2.20 | 4.4 | 1.27E-04 | LC-MS |
| 142 | **DL-Glyceraldehyde 3-phosphate** | 2.20 | 4.4 | 1.46E-08 | LC-MS |
| 143 | **L-Cystathionine** | 2.15 | 4.3 | 1.28E-02 | LC-MS |
| 144 | Cys-Gly | 2.12 | 4.24 | 1.98E-03 | LC-MS |
| 145 | N-acetyl-(L)-arginine | 2.09 | 4.18 | 1.33E-04 | LC-MS |
| 146 | N-Acetyl-D-fucosamine | 2.03 | 4.06 | 8.88E-04 | LC-MS |
| 147 | Aminopropylcadaverine | 2.01 | 4.02 | 6.77E-07 | LC-MS |
| 148 | **Glutathione disulfide** | 1.99 | 3.98 | 1.34E-05 | LC-MS |
| 149 | **L-2-Aminoadipate** | 1.94 | 3.88 | 1.42E-04 | LC-MS |
| 150 | (R)-S-Lactoylglutathione | 1.93 | 3.86 | 9.10E-03 | LC-MS |
| 151 | pyrophosphate | 1.91 | 3.82 | 3.05E-03 | LC-MS |
| 152 | 2-Hydroxyethanesulfonate | 1.90 | 3.8 | 2.44E-03 | LC-MS |
| 153 | **L-Asparagine** | 1.90 | 3.8 | 8.75E-05 | LC-MS |
| 154 | **GMP** | 1.88 | 3.76 | 1.68E-06 | LC-MS |
| 155 | Oleic acid | 1.84 | 3.68 | 3.57E-01 | GC-MS |
| 156 | beta-Alanine | 1.84 | 3.68 | 4.79E-03 | LC-MS |
| 157 | **Guanine** | 1.83 | 3.66 | 2.80E-06 | LC-MS |
| 158 | **NAD+** | 1.82 | 3.64 | 2.96E-04 | LC-MS |
| 159 | 3-oxo-5S-amino-hexanoic acid | 1.81 | 3.62 | 1.61E-03 | LC-MS |
| 160 | **Sucrose** | 1.80 | 3.6 | 1.00E-02 | LC-MS |
| 161 | N2-(D-1-Carboxyethyl)-L-arginine | 1.80 | 3.6 | 1.50E-02 | LC-MS |
| 162 | succinamate | 1.79 | 3.58 | 9.81E-04 | LC-MS |
| 163 | Fructose | 1.79 | 3.58 | 1.83E-03 | GC-MS |
| 164 | Xanthosine 5'-phosphate | 1.77 | 3.54 | 1.72E-02 | LC-MS |
| 165 | N6-Methyl-L-lysine | 1.75 | 3.5 | 2.56E-03 | LC-MS |
| 166 | [FA trihydroxy(4:0)] 2,3,4-trihydroxy-butanoic acid | 1.75 | 3.5 | 5.00E-02 | LC-MS |
| 167 | **Guanosine** | 1.72 | 3.44 | 4.32E-04 | LC-MS |
| 168 | **S-Adenosyl-L-methionine** | 1.72 | 3.44 | 4.04E-05 | LC-MS |
| 169 | Asp-Gly | 1.68 | 3.36 | 4.82E-04 | LC-MS |
| 170 | Mannose | 1.67 | 3.34 | 7.36E-03 | GC-MS |
| 171 | LysoPC(17:0) | 1.66 | 3.32 | 1.60E-02 | LC-MS |
| 172 | 1-Oleoylglycerophosphocholine | 1.65 | 3.3 | 2.65E-02 | LC-MS |
| 173 | Glycyl-leucine | 1.63 | 3.26 | 6.03E-05 | LC-MS |
| 174 | **L-Tryptophan** | 1.62 | 3.24 | 3.93E-03 | LC-MS |
| 175 | **D-Fructose 1,6-bisphosphate** | 1.61 | 3.22 | 5.77E-05 | LC-MS |
| 176 | Glu-Met | 1.60 | 3.2 | 6.79E-03 | LC-MS |
| 177 | **L-Tyrosine** | 1.60 | 3.2 | 2.25E-02 | LC-MS |
| 178 | **Adenosine** | 1.59 | 3.18 | 2.09E-03 | LC-MS |
| 179 | Glucopyranose | 1.59 | 3.18 | 2.94E-01 | LC-MS |
| 180 | 2,7-Anhydro-alpha-N-acetylneuraminic acid | 1.58 | 3.16 | 9.48E-04 | LC-MS |
| 181 | **S-glutathionyl-L-cysteine** | 1.57 | 3.14 | 3.31E-01 | LC-MS |
| 182 | Trehalose | 1.55 | 3.1 | 6.67E-02 | GC-MS |
| 183 | Glu-Glu-Met | 1.55 | 3.1 | 1.02E-02 | LC-MS |
| 184 | palmitic acid | 1.55 | 3.1 | 3.72E-01 | LC-MS |
| 185 | 3-Hydroxy-L-kynurenine | 1.54 | 3.08 | 2.01E-02 | LC-MS |
| 186 | talose | 1.53 | 3.06 | 1.23E-01 | LC-MS |
| 187 | **Allantoin** | 1.53 | 3.06 | 7.03E-03 | LC-MS |
| 188 | [PC (18:0)] 1-octadecanoyl-sn-glycero-3-phosphocholine | 1.53 | 3.06 | 5.89E-02 | LC-MS |
| 189 | **Inosine** | 1.53 | 3.06 | 1.45E-01 | LC-MS |
| 190 | Hypotaurine | 1.52 | 3.04 | 7.01E-03 | LC-MS |
| 191 | N-Methylnicotinate | 1.52 | 3.04 | 7.37E-02 | LC-MS |
| 192 | L-Glutamate 5-semialdehyde | 1.51 | 3.02 | 7.86E-04 | LC-MS |
| 193 | Hexadecasphinganine | 1.47 | 2.94 | 4.54E-02 | LC-MS |
| 194 | **5'-Methylthioadenosine** | 1.46 | 2.92 | 1.83E-01 | LC-MS |
| 195 | **N-methyl glucamine** | 1.46 | 2.92 | 1.63E-01 | LC-MS |
| 196 | (R)-2-Hydroxyglutarate | 1.45 | 2.9 | 4.72E-02 | LC-MS |
| 197 | **Deoxyadenosine** | 1.44 | 2.88 | 1.83E-01 | LC-MS |
| 198 | L-pyroglutamic acid | 1.44 | 2.88 | 8.91E-02 | GC-MS |
| 199 | **L-Proline** | 1.42 | 2.84 | 4.43E-02 | LC-MS |
| 200 | **L-Cystine** | 1.42 | 2.84 | 4.56E-01 | LC-MS |
| 201 | [PC (18:2)] 1-octadecadienoyl-sn-glycero-3-phosphocholine | 1.41 | 2.82 | 6.86E-02 | LC-MS |
| 202 | Thr-Asp-Ser | 1.40 | 2.8 | 6.09E-03 | LC-MS |
| 203 | **L-Phenylalanine** | 1.40 | 2.8 | 2.19E-02 | LC-MS |
| 204 | Mannitol | 1.39 | 2.78 | 3.58E-01 | GC-MS |
| 205 | Phosphoribosyl-AMP | 1.39 | 2.78 | 9.82E-02 | LC-MS |
| 206 | **L-Lysine** | 1.38 | 2.76 | 1.62E-02 | LC-MS |
| 207 | **L-Kynurenine** | 1.38 | 2.76 | 5.58E-02 | LC-MS |
| 208 | Trimethylamine N-oxide | 1.36 | 2.72 | 2.34E-03 | LC-MS |
| 209 | Malonate | 1.36 | 2.72 | 1.18E-01 | LC-MS |
| 210 | Ala-Asp-Ser | 1.36 | 2.72 | 9.62E-02 | LC-MS |
| 211 | **Adenine** | 1.35 | 2.7 | 2.24E-01 | LC-MS |
| 212 | 2-Naphthylamine | 1.35 | 2.7 | 4.76E-02 | LC-MS |
| 213 | Methyloxaloacetate | 1.34 | 2.68 | 1.31E-02 | LC-MS |
| 214 | L-Methionine S-oxide | 1.34 | 2.68 | 1.21E-03 | LC-MS |
| 215 | (S)-Methylmalonate semialdehyde | 1.34 | 2.68 | 2.26E-02 | LC-MS |
| 216 | Asp-Ser-Ser | 1.34 | 2.68 | 2.95E-01 | LC-MS |
| 217 | Mannonic acid | 1.34 | 2.68 | 8.58E-02 | LC-MS |
| 218 | **N6,N6,N6-Trimethyl-L-lysine** | 1.32 | 2.64 | 1.66E-01 | LC-MS |
| 219 | **Uracil** | 1.32 | 2.64 | 3.34E-04 | LC-MS |
| 220 | D-Galactofuranose | 1.31 | 2.62 | 8.79E-02 | LC-MS |
| 221 | n-Pentadecanoic acid | 1.31 | 2.62 | 9.48E-02 | LC-MS |
| 222 | L-cysteine sulfinate | 1.31 | 2.62 | 1.16E-01 | LC-MS |
| 223 | O-Palmitoyl-R-carnitine | 1.30 | 2.6 | 1.69E-01 | LC-MS |
| 224 | Creatine | 1.30 | 2.6 | 8.74E-02 | LC-MS |
| 225 | **L-Threonine** | 1.30 | 2.6 | 1.43E-02 | LC-MS |
| 226 | Xylitol | 1.30 | 2.6 | 1.91E-03 | LC-MS |
| 227 | Glu-Met-Thr | 1.26 | 2.52 | 2.80E-01 | LC-MS |
| 228 | tetracosahexaenoic acid | 1.22 | 2.44 | 1.62E-01 | LC-MS |
| 229 | Methylmalonate | 1.21 | 2.42 | 1.00E-01 | LC-MS |
| 230 | alpha-ketoglutaric acid | 1.20 | 2.4 | 2.01E-01 | GC-MS |
| 231 | **L-Glutamine** | 1.20 | 2.4 | 8.17E-02 | LC-MS |
| 232 | Tyramine | 1.19 | 2.38 | 2.40E-01 | LC-MS |
| 233 | N-Methylethanolamine phosphate | 1.18 | 2.36 | 4.96E-01 | LC-MS |
| 234 | **L-Alanine** | 1.17 | 2.34 | 2.07E-01 | LC-MS |
| 235 | Arg-Gln-Ser-Ser | 1.17 | 2.34 | 4.77E-01 | LC-MS |
| 236 | L-1-Pyrroline-3-hydroxy-5-carboxylate | 1.16 | 2.32 | 8.01E-02 | LC-MS |
| 237 | Creatinine | 1.15 | 2.3 | 2.32E-01 | LC-MS |
| 238 | Maltose | 1.13 | 2.26 | 4.76E-01 | LC-MS |
| 239 | 3-Oxopropanoate | 1.12 | 2.24 | 1.02E-01 | LC-MS |
| 240 | **L-serine** | 1.11 | 2.22 | 1.58E-01 | LC-MS |
| 241 | 5-6-Dihydrouridine | 1.11 | 2.22 | 2.74E-01 | LC-MS |
| 242 | Ethyl (R)-3-hydroxyhexanoate | 1.10 | 2.2 | 4.66E-01 | LC-MS |
| 243 | Cortisone | 1.10 | 2.2 | 7.89E-01 | GC-MS |
| 244 | **D-glucose** | 1.10 | 2.2 | 3.26E-01 | LC-MS |
| 245 | Methylimidazoleacetic acid | 1.10 | 2.2 | 3.58E-01 | LC-MS |
| 246 | **Phenylacetylglycine** | 1.10 | 2.2 | 5.97E-01 | LC-MS |
| 247 | D-Galactose | 1.09 | 2.18 | 6.83E-01 | LC-MS |
| 248 | **3-Phosphoglycerate** | 1.09 | 2.18 | 4.29E-01 | LC-MS |
| 249 | Propanoic acid | 1.08 | 2.16 | 5.91E-01 | LC-MS |
| 250 | Sulfoacetaldehyde | 1.08 | 2.16 | 6.24E-01 | LC-MS |
| 251 | Homocysteine | 1.08 | 2.16 | 7.51E-01 | LC-MS |
| 252 | **Orthophosphate** | 1.06 | 2.12 | 8.22E-01 | LC-MS |
| 253 | 10-Hydroxydecanoic acid | 1.05 | 2.1 | 4.54E-01 | LC-MS |
| 254 | Glycodeoxycholate | 1.05 | 2.1 | 7.75E-01 | LC-MS |
| 255 | Dodecatetraenedioic acid | 1.05 | 2.1 | 6.00E-01 | LC-MS |
| 256 | 2-C-Methyl-D-erythritol 4-phosphate | 1.04 | 2.08 | 9.17E-01 | LC-MS |
| 257 | Cys-Cys-His-His | 1.04 | 2.08 | 7.96E-01 | LC-MS |
| 258 | **phosphoenolpyruvic acid** | 1.03 | 2.06 | 8.48E-01 | LC-MS |
| 259 | 5-Hydroxyindoleacetate | 1.02 | 2.04 | 8.26E-01 | LC-MS |
| 260 | Phe-Asp | 1.01 | 2.02 | 9.49E-01 | LC-MS |
| 261 | phosphoric acid | 1.01 | 2.02 | 9.57E-01 | LC-MS |
| 262 | N-Acetyl-D-glucosamine 6-phosphate | 1.00 | 2 | 9.84E-01 | LC-MS |
| 263 | Glu-Leu-Thr-His | -1.01 | -2.02 | 9.61E-01 | LC-MS |
| 264 | His-Phe-Val-Pro | -1.01 | -2.02 | 9.66E-01 | LC-MS |
| 265 | **Phenylpyruvate** | -1.02 | -2.04 | 8.64E-01 | LC-MS |
| 266 | hydroxy-octadecadienoic acid | -1.03 | -2.06 | 8.40E-01 | LC-MS |
| 267 | L-Noradrenaline | -1.03 | -2.06 | 8.39E-01 | LC-MS |
| 268 | di-n-Undecylamine | -1.03 | -2.06 | 9.42E-01 | LC-MS |
| 269 | Fructoselysine | -1.04 | -2.08 | 6.75E-01 | LC-MS |
| 270 | Acetamide, N,N-diethyl- | -1.05 | -2.1 | 3.12E-01 | GC-MS |
| 271 | 3-Butenoic acid | -1.05 | -2.1 | 1.99E-01 | LC-MS |
| 272 | glycolic acid | -1.05 | -2.1 | 3.77E-01 | LC-MS |
| 273 | Myo-inositol-3-phosphate | -1.05 | -2.1 | 8.44E-01 | GC-MS |
| 274 | Erucic acid | -1.06 | -2.12 | 7.49E-01 | GC-MS |
| 275 | 5-Hydroxypentanoate | -1.07 | -2.14 | 4.88E-01 | LC-MS |
| 276 | [FA (20:4)] 5Z,8Z,11Z,14Z-eicosatetraenoic acid | -1.07 | -2.14 | 6.04E-01 | LC-MS |
| 277 | Cholest-2-eno[2,3-b]indole, 1'-acetyl-6'-methoxy- | -1.08 | -2.16 | 5.28E-01 | GC-MS |
| 278 | N3-methylcytosine | -1.08 | -2.16 | 6.50E-01 | LC-MS |
| 279 | D-Threose | -1.08 | -2.16 | 4.19E-01 | LC-MS |
| 280 | 4-Methylene-L-glutamine | -1.09 | -2.18 | 6.82E-01 | LC-MS |
| 281 | 2-Phenylacetamide | -1.09 | -2.18 | 5.21E-01 | LC-MS |
| 282 | Pyruvate | -1.09 | -2.18 | 1.13E-03 | GC-MS |
| 283 | Heptanedioic acid | -1.10 | -2.2 | 4.02E-01 | LC-MS |
| 284 | methyl-dihydroxy-pentanoic acid | -1.12 | -2.24 | 3.39E-01 | LC-MS |
| 285 | dioxo-octanoic acid | -1.13 | -2.26 | 3.67E-01 | LC-MS |
| 286 | amino-undecanoic acid | -1.14 | -2.28 | 3.49E-01 | LC-MS |
| 287 | **L-Citrulline** | -1.17 | -2.34 | 5.76E-01 | LC-MS |
| 288 | **sn-glycero-3-Phosphocholine** | -1.17 | -2.34 | 7.07E-01 | LC-MS |
| 289 | Methanesulfonic acid | -1.18 | -2.36 | 5.11E-01 | LC-MS |
| 290 | Tetradecanedioic acid | -1.18 | -2.36 | 3.52E-01 | LC-MS |
| 291 | P-DPD | -1.18 | -2.36 | 2.76E-01 | LC-MS |
| 292 | Phthalic acid | -1.19 | -2.38 | 6.18E-03 | LC-MS |
| 293 | 4-Acetamidobutanoate | -1.19 | -2.38 | 1.49E-01 | LC-MS |
| 294 | myo-Inositol | -1.20 | -2.4 | 4.94E-01 | LC-MS |
| 295 | Cyclododecane | -1.20 | -2.4 | 3.07E-02 | GC-MS |
| 296 | Gamma-Glutamylglutamine | -1.21 | -2.42 | 1.26E-01 | LC-MS |
| 297 | N4-acetyl-N4-hydroxy-1-aminopropane | -1.22 | -2.44 | 4.86E-01 | LC-MS |
| 298 | 5-oxo-7-octenoic acid | -1.22 | -2.44 | 1.24E-01 | LC-MS |
| 299 | 2-Acetolactate | -1.24 | -2.48 | 3.78E-01 | LC-MS |
| 300 | Urea | -1.26 | -2.52 | 4.61E-01 | GC-MS |
| 301 | Elaidiccarnitine | -1.26 | -2.52 | 2.77E-01 | LC-MS |
| 302 | d-Xylose | -1.27 | -2.54 | 4.61E-01 | LC-MS |
| 303 | D-Glycerate | -1.28 | -2.56 | 9.06E-02 | LC-MS |
| 304 | **Lactate** | -1.29 | -2.58 | 2.85E-01 | LC-MS |
| 305 | 2-acetamidoglucal | -1.30 | -2.6 | 7.17E-03 | LC-MS |
| 306 | Heme | -1.32 | -2.64 | 3.71E-01 | LC-MS |
| 307 | D-4'-Phosphopantothenate | -1.35 | -2.7 | 5.89E-02 | LC-MS |
| 308 | **Glycerol** | -1.39 | -2.78 | 1.43E-03 | LC-MS |
| 309 | Met-Ala-Gly | -1.40 | -2.8 | 6.53E-02 | LC-MS |
| 310 | Urate | -1.41 | -2.82 | 1.34E-01 | LC-MS |
| 311 | Stearic acid | -1.44 | -2.88 | 8.78E-02 | GC-MS |
| 312 | 4-Guanidinobutanoate | -1.44 | -2.88 | 3.24E-03 | LC-MS |
| 313 | Deoxycytidine | -1.47 | -2.94 | 1.75E-01 | LC-MS |
| 314 | 2-monooleoylglycerol | -1.53 | -3.06 | 1.16E-02 | LC-MS |
| 315 | 6-[3]-ladderane-1-hexanol | -1.56 | -3.12 | 9.22E-02 | LC-MS |
| 316 | 4-Hydroxy-L-threonine | -1.59 | -3.18 | 1.30E-05 | LC-MS |
| 317 | Val-Asp-Gly | -1.60 | -3.2 | 1.09E-04 | LC-MS |
| 318 | Ala-Ser | -1.64 | -3.28 | 3.31E-04 | LC-MS |
| 319 | Heptadecanoic acid | -1.64 | -3.28 | 4.55E-02 | GC-MS |
| 320 | Leu-Ala | -1.76 | -3.52 | 1.04E-04 | LC-MS |
| 321 | 9,12-octadecadienal | -1.79 | -3.58 | 1.70E-01 | LC-MS |
| 322 | Thr-Ala-Asp | -1.80 | -3.6 | 9.52E-05 | LC-MS |
| 323 | N-Acetyl-D-glucosaminate | -1.94 | -3.88 | 1.55E-05 | LC-MS |
| 324 | D-Sorbitol | -2.11 | -4.22 | 8.20E-09 | LC-MS |
| 325 | **L-Histidine** | -2.40 | -4.8 | 2.99E-08 | LC-MS |
| 326 | N-Ribosylnicotinamide | -2.42 | -4.84 | 1.97E-03 | LC-MS |
| 327 | octadecenamide | -2.76 | -5.52 | 1.60E-01 | LC-MS |
| 328 | Valine | -2.85 | -5.7 | 2.69E-03 | GC-MS |
| 329 | Disaccharide | -2.95 | -5.9 | 9.12E-02 | GC-MS |
| 330 | N5-(L-1-Carboxyethyl)-L-ornithine | -3.10 | -6.2 | 1.01E-08 | LC-MS |
| 331 | Hexose phosphate | -3.55 | -7.1 | 5.65E-10 | LC-MS |
| 332 | 3-beta-D-Galactosyl-sn-glycerol | -5.38 | -10.76 | 1.68E-06 | LC-MS |
| 333 | Leu-Lys-Asp | -5.64 | -11.28 | 1.04E-08 | LC-MS |
