# Supplementary material for: Host Reticulocytes Provide Metabolic Reservoirs That Can Be Exploited by Malaria Parasites
Source: PLoS Pathog. 2015 Jun 4;11(6):e1004882. doi: 10.1371/journal.ppat.1004882 (PMC4456406; doi:10.1371/journal.ppat.1004882)
Supplement: S2 Table — (DOCX) [file ppat.1004882.s003.docx]

# Table S2

List of primers.

| Primer | Sequence | Comments |
| --- | --- | --- |
| GU2051 | GATAATGTCCTACTTTTTCTTTG | *pepc* 5’homology arm forward |
| GU2052 | TATATAGCTGCTTGAGACAC | *pepc* 5’ homology arm reverse |
| GU2053 | GCAAAATACCGGATAACTC | *pepc* 3’homology arm forward |
| GU2054 | TTTAGGAAACCAATCAAAGAG | *pepc* 3’ homology arm reverse |
| GU2057 | GGGCTTTATACTATTTTTTTGTC | *pepc* ko 5’ integration forward |
| GU2058 | TATCGTGGTAGAGTAAAACTG | *pepc* ko 3’ integration reverse |
| GU2059 | CATGATTTATCCGAAAAATATAGTG | *pepc* orf forward |
| GU2060 | GTGCTTTATATACATATACAACAC | *pepc* orf reverse |
| GU2198 | GGAATTATAATTCTTAACCCTAACATTTTAACCTCTC | *mdh* 5’homology arm forward |
| GU2199 | CTTGTCGTATATGCACTCGGTGTTGG | *mdh* 5’ homology arm reverse |
| GU2200 | CCTTAAAATGGATAGTCAAATTGATCGTACACAACTAA | *mdh* 3’homology arm forward |
| GU2201 | CATCTCTAATTCGTTAGAATTTATTATAGACTACG | *mdh* 3’ homology arm reverse |
| GU2278 | CCACTGTAATCATAGAACAGTTCAACTAC | *mdh* ko 5’ integration forward |
| GU2279 | CAAGATTAGTACACATTGGATTAATGGG | *mdh* ko 3’ integration reverse |
| GU2280 | CATTAATAGGAAGTGGCCAAATAGGG | *mdh* orf forward |
| GU2281 | GATAGCAAGCTTGTTCTTCTTCTGTC | *mdh* orf reverse |
| GU2190 | CCTTTTCCTTTTGTTTTATCCATCCATTTA | *oprt* 5’homology arm forward |
| GU2191 | AATCTCAAATTGTGAAATAAACAATAAAAAATTTTGTC | *oprt* 5’ homology arm reverse |
| GU2192 | CTGAGTTCTGTATTTACTTTCATAAGTTTTTAAACG | *oprt* 3’homology arm forward |
| GU2193 | CCCACATAAGTAAATATACATACACATATTATTATGC | *oprt* 3’ homology arm reverse |
| GU2286 | CTTAAATTAGCATTACTGCGTACATCCC | *oprt* ko 5’ integration forward |
| GU2610 | GAGCTAGCTGAAAGTTGCAAT | *oprt* ko 3’ integration reverse |
| GU2288 | GATGAAGAATTACACAAAAAATACAATGAATTATGC | *oprt* orf forward |
| GU2289 | GTGAAATATCTTCTTCATAATTAAGGATGC | *oprt* orf reverse |
| GU2194 | GATGCTCTCTCGTATATCCGTTTAAATTAC | *ompdc* 5’homology arm forward |
| GU2195 | GCTAGCTATGAATTTTAGTTGATAGATTTTTTATTTG | *ompdc* 5’ homology arm reverse |
| GU2196 | GAATACATTGAGTTTAACGGAACTCAATTTAATAGCC | *ompdc* 3’homology arm forward |
| GU2197 | GCATGCAATATTGGCAATACATGAAAACGAATTAATAT | *ompdc* 3’ homology arm reverse |
| GU2282 | GCACCCATATTTATATCAACATTTCTATCAG | *ompdc* ko 5’ integration forward |
| GU2283 | GCACAATTTTACATATCGATATATGTACAATG | *ompdc* ko 3’ integration reverse |
| GU2284 | GTATTGGGTTGGATCCTGATGAAG | *ompdc* orf forward |
| GU2285 | CTTGTTCAATATTACCACCATTTTCTATGTC | *ompdc* orf reverse |
| GU2061 | GTAAACTTAAGCATAAAGAGCTCG | 5’ integration reverse (in plasmid) |
| GU0204 | GTCTCTTCAATGATTCATAAATAG | 3’ integration forward (in plasmid) |
