# Supplementary material for: Host Reticulocytes Provide Metabolic Reservoirs That Can Be Exploited by Malaria Parasites
Source: PLoS Pathog. 2015 Jun 4;11(6):e1004882. doi: 10.1371/journal.ppat.1004882 (PMC4456406; doi:10.1371/journal.ppat.1004882)
Supplement: S1 Supplementary Materials and Methods — (DOCX) [file ppat.1004882.s005.docx]

Supplementary materials and methods

## Rodent erythrocyte sample preparation for metabolomics

For collecting cells of Reticulocyte enriched Erythrocyte Population (REP), 3 female Wistar rats were each injected with phenylhydrazine-HCl dissolved in 0.9% NaCl (w/v) at a dose of 100 mg/kg body weight and reticulocyte percentage in peripheral blood was monitored using reticulocyte marker CD71-APC antibody by FACS analysis performed on a MACSQuant analyser (Miltenyi Biotec, Germany). On day 5, all rats showed reticulocyte population to be around 30-35% and were bled by cardiac puncture and blood from each rat was collected in RPMI1640 medium. Cells of wild type Erythrocyte Population (wtEP) (with ~1.5% reticulocytes) were prepared similarly without phenylhydrazine-HCl treatment. Each suspension was quickly passed through a prewashed Plasmodipur filter to remove leucocytes and eluted with RPMI1640 medium. A thermometer was put in the bottom of the suspension and the tube submerged in a dry-ice ethanol bath with gentle agitation until the temperature reached 8˚C, at which point the tube was immediately immersed in an ice bucket. Using this protocol, the temperature of the cell suspension reached 0^o^C within 10-12 seconds. The chilled suspension was centrifuged at 450 g for 8 mins, supernatant was removed and the volumes of equal number of reticulocyte enriched and un-enriched erythrocytes were found to be the same. The cell pellets were resuspended in ice-cold enriched PBS (containing 20 mM Hepes, 20 mM Glucose, 4 mM NaHCO_3_, and 0.1% BSA). The cell density of the suspension was determined using a haemocytometer and replicates containing 1 x 10^8^ cells were prepared in enriched PBS (up to 2ml per tube) and kept on ice until metabolite extraction. After first wash (centrifugation at 4˚C for 10 mins at 1,300g) the pellet was resuspended with 500 µl cold enriched PBS for the second wash (centrifugation at 4˚C for 5 mins at 2,700g) and supernatant was removed again. Finally, the washed pellets were suspended in cold 200 µl of chloroform/methanol/water (1:3:1 v/v) containing internal standards (5-fluorouridine, Cl-phenyl-cAMP, N-methyl glucamine, canavanine and piperazine) at 1 µM concentration. After vigorous mixing in a cooled (4˚C) shaker for 1 hour and sonication (2 mins, 0°C), the suspension was centrifuged (at 4°C for 5 mins at 15,300 g) and equal aliquots (90 µl) of the supernatant were transferred to separate tubes for LC-MS and GC-MS analyses. Pooled sample QCs were prepared at this stage for quality control during LC-MS and GC-MS analysis. Tubes for LC-MS analyses were topped up with nitrogen, capped tightly and kept at -80˚C before running. Tubes for GC-MS analyses were dried down under nitrogen flow, capped tightly and put at -80˚C before reconstitution with chloroform/methanol/water (1:3:1 v/v) prior to derivatization. All experiments were performed according to the Home Office licence regulations and the local ethical committees.

## Metabolomic analysis of rodent erythrocytes

For LC-MS analysis, samples underwent hydrophilic interaction liquid chromatography (HILIC) (UltiMate 3000 RSLC, Thermo Fisher) with a 20mm x 2.1mm ZIC-HILIC guard column coupled to a 150 x 4.6mm ZIC-HILIC analytical column running at 300 µl/min, coupled to an Exactive Orbitrap mass spectrometer (Thermo Fisher). The LC-MS method is based on a previously published HILIC method [[1](#_ENREF_1)] with a gradient starting at 20% H_2_O with 0.1% formic acid (A) and 80% acetonitrile with 0.1% formic acid (B), decreasing to 20% B at 30 mins, followed by a wash at 5% B for 6 mins, and equilibration at 80% B for 8 mins. Raw mass spectrometry data was processed using the standard Glasgow Polyomics pipeline, consisting of XCMS for peak picking [[2](#_ENREF_2)], MzMatch for filtering and grouping [[3](#_ENREF_3)] and IDEOM for further filtering, post-processing and identification [[4](#_ENREF_4)]. Core metabolite identifications were validated against a panel of unambiguous standards by mass and retention time. Additional putative identifications were assigned by mass and predicted retention time [[1](#_ENREF_1)] . Automatic metabolite identification was followed by manual data filtration for removing false positives and duplicate identifications and including false rejections.

For GC-MS, dried samples were suspended in chloroform/methanol/water (1:3:3 v/v) and biphasic partitioning was carried out by centrifugation and pipetting out the upper methanol-water phase containing mostly polar metabolites which was retained for this study. Some apolar metabolites were also detected in this fraction as some charged free fatty acids preferentially partition into this phase. Samples were dried under nitrogen and subjected to automated methoximation and TMS derivatisation using an Gerstal autosampler/sample preparation robot fitted to an Agilent- 7890A GC-5975 MSD instrument. Briefly, samples were methoximated in 20 mg/ml methoxyamine in pyridine (20 µl ) with shaking at 37°C for 2 hours and then derivitized by addition of BSTFA + 1% TMCS (20 µl) silylation reagent and shaking at 37°C for 1 hr. After incubation at room temperature for 1 hour, sample (1 µl) was analyzed on an Agilent 7890A GC -5975 C mass-detector instrument, equipped with a VF5-MS column (30 m, 0.25 mm inner diameter) and helium as the carrier gas. The oven temperature was held at 70°C (1min), then ramped at 1°C/min to 76°C, then 5°C/min to 325°C and held for 10 mins. GC/MS peaks were aligned using the Metabolomics software PyMS [[5](#_ENREF_5)] which generated a data matrix of candidate metabolites showing their intensity representing abundance of a metabolite in a given sample and its unique retention time. Chromatograms were manually checked using Agilent Chemstation software and the peaks corresponding to the retention times in the PyMS matrix were identified based on their Electron Ionisation (EI) spectrum. Metabolites were assigned putative identities by matching their spectra (with a cut-off score of ≥90%) to Agilent Fiehn and NIST GC-MS Metabolomics libraries of metabolite GC-MS spectra.

From a total number of 4560 peaks collected from the two platforms, 333 metabolites were putatively annotated in erythrocytes, with identification in LC-MS data based on accurate mass and predicted retention time (minimum confidence value of 5/10 in IDEOM) [[4](#_ENREF_4)] and GC-MS based on spectral matching to Agilent Fiehn [[6](#_ENREF_6)] and NIST (National Institute of Standards and Technology, Gaithersburg, MD, USA) spectral libraries (minimum spectral match score of 90% in Chemstation). It is noted that many more probable metabolites were detected but were not readily assigned an identity according to these parameters. From all 333 metabolites, 293 metabolites were identified by LC-MS and 40 metabolites were identified by GC-MS, with 17 metabolites identified by both platforms (Table S1). Of the overlapping metabolites measured on both platforms, the fold-change values in abundance observed between REP and wtEP were found to be consistent in both direction and magnitude on both platforms. These metabolites were quantitated using the LC-MS data in order to avoid duplication. All raw data is available from the authors on request.

## Metabolomics analysis of Human CD34+ stem cell grown erythrocytes

Peripheral blood mononucleated cells were obtained from blood by Percoll density purification and CD34+ hemopoietic progenitor cells were isolated by magnetic bead separation according to the manufacturer's instructions (Miltenyi Biotec). CD34+ cells were cultured in a three-stage protocol based on the methods of [[7](#_ENREF_7)]. Initially cells were cultured at 37°C in a humid atmosphere of 5% CO2 at a density of 1 x 10^4^ cells/mL and then maintained in the range of 2-10 × 10^5^ cells/mL in IMDM (LifeTech) containing 5% (v/v) AB Serum (Interstate Companies Laboratories), 10μg/ml Insulin (Sigma), 3U/ml heparin (Pfizer), 200μg/ml Transferrin (Prospec), 3U/ml EPO (Eprex). During stage one (days 0-8) this was supplemented with 10ng/ml SCF (GenScript) and 1ng/ml IL-3 (R&D systems); during stage two (days 8-11) with 10ng/ml SCF and additional 800μg/ml transferrin and stage 3 (days 11-18) with 3U/ml EPO and additional 800 μg/ml transferrin. Cultured reticulocytes (cRetics) were filtered at day 18 using a PALL WBF leukocyte filter. Isogenic control red blood cells (RBCs) were retained from donor blood, washed in IMDM and stored in saline-adenine-glucose-mannitol solution (SAG-M) at 4°C prior to use. Cells were washed and cultured overnight in stage 3-supplemented IMDM (as outlined above). Metabolism was quenched by immersion of cultures in an ethanol/dry ice bath to 0-4 °C. Cells were pelleted by centrifugation (10,000 rpm for 1 min) and washed in cold PBS (1 mL). Metabolites were extracted from 1 x 10^8^ RBCs and 0.5 x 10^8^ cRetics by addition of 300 µL chloroform/methanol/water (1:3:1 v/v) containing internal standards (CHAPS, CAPS, PIPES and TRIS; 1 µM) and left for 30 mins at 4 °C with periodic mixing and sonication. Cellular debris was removed by centrifugation (16,000 rpm for 10 mins) and the supernatant was dried under nitrogen gas prior to analysis. Samples were reconstituted in 80% acetonitrile for analysis by high resolution LC-MS with minor adjustments to the previously described ZIC-pHILIC-AC method [[8](#_ENREF_8)]. The chromatography utilized a 4.6 x 150 mm, 5 µm ZIC-pHILIC column (Sequant) with 10 mM ammonium carbonate (A) and acetonitrile (B) mobile phase at a flow rate of 300 µL/min. The gradient ran from 80% B (0 min) to 40% B (20 mins), then to 5% B for 3 mins (23-26 mins) followed by re-equilibration at 80% B for 9 mins (29-38 mins). Mass detection was performed on an Agilent Q-TOF 6550 (Agilent Technologies) operating in negative mode electrospray ionisation with capillary voltage 3.5 kV and fragmenter voltage 175 V. Initial data analysis was conducted with the IDEOM package as described above [[4](#_ENREF_4)] and peak areas for selected metabolites were extracted based on accurate mass and retention time and manually verified with the MassHunter software. Comparison of the average and median peak heights from all detected LC-MS peaks demonstrated no significant difference between reticulocytes and mature erythrocytes, confirming that the cell number based normalization, with adjustment for cell size (i.e. twice as many mature erythrocytes than reticulocytes per sample), was appropriate. Two experiments were performed in triplicate, using cells (cRetics and corresponding isogenic RBCs) from two independent donors.

## Infection of laboratory animals with *P. berghei* parasites

For mouse infections, female Theilers Original (TO) outbred mice of body weight 26-30g were used. Cryopreserved blood stages were thawed at room temperature and 0.02-0.5 ml of the suspension was injected intraperitoneally into a mouse. For mouse infection with blood stages obtained from an infected mouse (mechanical passage), one droplet of tail blood (5 µl) was collected from an infected animal with a parasitemia of 5-15% in 10 ml PBS and 0.1 ml of the suspension was injected intraperitoneally into a mouse. On day 4-7 after injection the parasitemia increased from 0.1 to 5-20%. For rat infection, female Wistar rats of body weight 150-175 g were used. To infect these, 5-8 droplets of tail blood (30-40 µl) were collected from an infected animal with a parasitemia of 5-15% in 1ml PBS and the 1ml suspension was injected intraperitoneally. On day 4 or 5 after injection the parasitemia ranged between 0.5-3%.

## Asexual cultures of *P. berghei*

*P. berghei* cultures were maintained for one cycle using standard methods. RPMI1640 (containing 5 g/L of Albumax II ®) were used as the growing medium and flasks were gassed for 30 seconds with a gas mix containing 5% CO_2_, 5% O_2_, 90% N_2_ and incubated overnight at 37˚C on a shaker at a minimal speed just to keep the cells in suspension. Maturation of schizonts and number of merozoites per schizonts were analysed in Giemsa stained smears made from *in vitro* cultures.

## Generation of knockout parasites and cloning

*P. berghei* schizonts (from line RMgm-7 which expresses GFP constitutively under *eef1a* promoter and from line RMgm-164 which expresses GFP in male gametocytes and RFP in female gametocytes) were transfected with linear DNA constructs containing the yfcu-hdhfr selectable marker flanked by homology arms (generated using primers in table S2) corresponding to 5’UTR and 3’UTR of the orf of the gene of interest respectively, injected intravenously in female Wistar rats and TO mice and selected by pyrimethamine in drinking water as described in [[9](#_ENREF_9)]. Resulting transfectants were analysed by PCR for 5’ and 3’ integration (using primers in table S2) , cloned by limiting dilution and the absence of open reading frame in the mutants confirmed by PCR. For further phenotypic analysis, due to reasons of cost effectiveness and ease of handling, all mutants generated in TO mice were used and experiments were done by obtaining parasites grown in TO mice.

## Asexual growth competition assay

Equal numbers of mutant parasites (10^6^ cells) made in RMgm-7 background expressing GFP constitutively under *eef1a* promoter were mixed with wt parasites (10^6^ cells) (RMgm- 86) expressing RFP under the same promoter and the mixture was injected into a mouse. The population of infected erythrocytes (iRBCs) was monitored by Hoechst staining and the proportion of iRBCs expressing GFP and RFP was recorded by FACS analysis over the course of two weeks. Infected blood from first mouse was sequentially passage into two or three mice to avoid multiple infections over this period.

## Lethality experiments in C57/B6 mice

iRBCs (10^4^) were injected intra-peritoneally into female 8-10 weeks old C57/B6 mice (n=5 per line) and parasitemia, disease pathology and mortality was monitored over 21 days.

## Gametocyte conversion monitoring by FACS during blood stage growth

Mutants made in the RMgm-164 background which expresses GFP in male gametocytes and RFP in female gametocytes along with wt were grown in mice and peripheral blood was monitored by FACS analysis by checking for infected erythrocytes (iRBCs) by Hoechst staining and the proportion of iRBCs expressing GFP and RFP, indicative of the presence of male and female gametocytes.

## Exflagellation assay and DNA quantification by FACS

During gametogenesis, male gametocytes undergo rapid endomitosis and DNA content is increased from 1n to 8n within 8 mins after activation and adherent clumps of erythrocytes are formed around the activating gametocytes called exflagellation centres which were counted on haemocytometer. DNA staining in exflagellating gametocytes was observed by fixing MACS-column purified activating gametocytes (devoid of asexual stages and leucocytes as infected mice were treated with 25mg/ml sulfadiazine in drinking water for 48 hours before harvesting and the blood suspension was passed through a pre-washed Plasmodipur filter) using 0.25% glutaraldehyde at 4 mins intervals, staining with 10 µM Hoechst 33258 dye in PBS for 1 h at 37°C and doing FACS analysis on a CyAn ADP Analyser. UV excitation of Hoechst 33258 dye was performed with a violet laser (405 nm) and the gametocyte population was selected by gating on forward/side light scatter. The fluorescence intensity of a total of 100,000 cells was measured for each sample. The mean fluorescence intensity of the activating gametocyte is proportional to the mean DNA content of the parasites and activating male gametocytes and female gametocytes were gated based on DNA content at different time points based on the wt control. All data was plotted normalised to the controls.

## Ookinete cultures of *P. berghei* and conversions

Mice infected with *P. berghei* were given sulfadiazine in drinking water which killed all asexual stage parasites in 48 hours and circulating gametocytes remain in blood. Mice were bled and infected blood was collected in RPMI1640 containing 5 g/L Albumax II® and 100 µM xanthurenic acid to activate gametocytes. Cultures were incubated at 21°C for 21 hours and giemsa smears were made for counting mature ookinetes and female gametes whose cumulative ratio to female gametes only gave the ookinete conversion rate.

## ***in vitro*** sexual crosses

Equal numbers of gametocytes from two *P. berghei* lines obtained from infected TO mice treated with sulfadiazine in drinking water were taken and mixed in activation media. The suspension was then incubated at 21°C for 21 hours and Giemsa smears were made for counting mature ookinetes and female gametes

## Mosquito transmission experiments

*P. berghei* infected mice with a parasitemia of 5-10% were used to blood feed a cage of 250 mosquitoes for 10 mins. Mature oocysts were counted in mosquito midguts between days 12-14 using a Leica M205 FA Fluorescence Stereomicroscope. Salivary gland sporozoites were checked between days 21-25. Infected mosquitoes were allowed to feed on naïve mice for 10 mins between days 21-25 and these mice were observed for parasites by making giemsa stained blood smears between days 3-14 to check for successful transmission.

## Determination of IC_50_ value of *P. berghei* inhibitors *in vitro*

Inhibitors were used to perform *in vitro* drug susceptibility tests in standard short-term cultures of synchronized *P. berghei* blood stages. Cultured and purified schizonts/merozoites of the reference ANKA strain of *P. berghei* line cl15cy1, obtained by Nycodenz density gradient purification were injected i.v. into the tail vein of a TO mouse. Injected merozoites invade within 4h after injection and newly infected blood was collected from the mouse by heart puncture at 4h after the injection of the purified schizonts/merozoites. Infected blood was washed once (450 g, 8 mins) with complete culture medium (RPMI1640 + 25% FCS, pH 7.5) followed by mixing of infected erythrocytes with serially diluted solutions of inhibitors in complete culture medium and incubated in 24-well plates in triplicate at a final parasitaemia of 1% at 37°C for 24h under special gas mix of 5% CO_2_, 5% O_2_, 90% N_2_, conditions that permit ring forms to develop into mature schizonts. Parasite development was analysed by FACS after staining iRBCs with DNA-specific dye Hoechst-33258. The cells were pelleted by centrifugation (450 g, 8 mins) and after removal of supernatant, cells were fixed with 0.25% glutaraldehyde/PBS solution and stained with 10 µM Hoechst-33258 solution in PBS for 1h at 37°C. Stained cells were analysed using MACSQuant analyser (Miltenyi Biotec, Germany). UV excitation of Hoechst-33258 dye was performed with a violet laser (450/50 nm) and the iRBC population was selected by gating on forward/sidelight scatter. A total of 100,000 cells per samples were analysed and mature schizonts were gated based on their fluorescence intensity and counted in each sample. For determination of growth inhibition, the number of mature schizonts observed was set to correspond to 100% growth for no drug controls and percentage growth was calculated accordingly for the drug treated samples. 100% growth values were in the range 60-75% conversion of ring stage parasites to schizonts (15-20% of ring stage parasites committed to making gametocytes do not undergo DNA replication). Growth inhibitory curves were constructed in Graph pad Prism and based on data from three independent repeats, the IC_50_ value for blood stage inhibition of *P. berghei* parasites were calculated. Giemsa stained smears from drug treated cultures were also checked to determine the stage at which parasites were growth arrested.

## Determination of IC_50_ value of *P. falciparum* asexual growth inhibition *in vitro*

*P. falciparum* 3D7 strain was used for determining IC_50_ values of inhibitors in *in vitro* cultures by measuring ^3^H-Hypoxanthine incorporation in the presence of inhibitors in increasing concentrations as described [[10](#_ENREF_10)]. Cultures were set up at 0.5 % parasitaemia and approximately 2% hematocrit in complete RPMI medium without hypoxanthine (IC_50_ medium). Human erythrocytes were washed and stored in RPMI1640 (without AlbumaxII®) at 4°C for not more than a week. A serial dilution of 2 times of the required inhibitor concentration was prepared in a 96 well plate in similar IC_50_ medium. In each well, 100 µl of inhibitor was mixed with 100 µl of cells, creating a 1 times final concentration of the inhibitor. Incorporation of ^3^H-hypoxanthine (1Ci/ ml, specific activity 20Ci/ mmol) in uninfected erythrocytes and parasites incubated without inhibitor was also measured as negative control. Plates were incubated for 48 hours at 37°C in the presence of a specialized gas mix (5% CO_2_, 1% O_2_, 94% N_2_). After 48 hours of incubation, 100 µl of medium from each well was replaced with fresh medium containing 0.4 µCi ^3^H-hypoxanthine per well. Plates were incubated for further 24 hours and then frozen at -20°C. Then the plates were defrosted and harvested using a Tomtec Mach III harvester and Wallac Printed Filter Mat- A filter mats. The filter mats were dried at 60°C for one hour and sealed in a plastic bag with 4 ml scintillation liquid. Radioactive decay was measured in a Wallac Trilux MicroBeta counter for 1 min per well. IC_50_ values were calculated using GraphPad Prism software.

# References

1. Creek DJ, Jankevics A, Breitling R, Watson DG, Barrett MP, et al. (2011) Toward global metabolomics analysis with hydrophilic interaction liquid chromatography-mass spectrometry: improved metabolite identification by retention time prediction. Anal Chem 83: 8703-8710.

2. Smith CA, Want EJ, O'Maille G, Abagyan R, Siuzdak G (2006) XCMS: processing mass spectrometry data for metabolite profiling using nonlinear peak alignment, matching, and identification. Anal Chem 78: 779-787.

3. Scheltema RA, Jankevics A, Jansen RC, Swertz MA, Breitling R (2011) PeakML/mzMatch: a file format, Java library, R library, and tool-chain for mass spectrometry data analysis. Anal Chem 83: 2786-2793.

4. Creek DJ, Jankevics A, Burgess KE, Breitling R, Barrett MP (2012) IDEOM: an Excel interface for analysis of LC-MS-based metabolomics data. Bioinformatics 28: 1048-1049.

5. O'Callaghan S, De Souza DP, Isaac A, Wang Q, Hodkinson L, et al. (2012) PyMS: a Python toolkit for processing of gas chromatography-mass spectrometry (GC-MS) data. Application and comparative study of selected tools. BMC Bioinformatics 13: 115.

6. Kind T, Wohlgemuth G, Lee do Y, Lu Y, Palazoglu M, et al. (2009) FiehnLib: mass spectral and retention index libraries for metabolomics based on quadrupole and time-of-flight gas chromatography/mass spectrometry. Anal Chem 81: 10038-10048.

7. Douay L, Giarratana MC (2009) Ex vivo generation of human red blood cells: a new advance in stem cell engineering. Methods Mol Biol 482: 127-140.

8. Zhang T, Creek DJ, Barrett MP, Blackburn G, Watson DG (2012) Evaluation of coupling reversed phase, aqueous normal phase, and hydrophilic interaction liquid chromatography with Orbitrap mass spectrometry for metabolomic studies of human urine. Anal Chem 84: 1994-2001.

9. Janse CJ, Ramesar J, Waters AP (2006) High-efficiency transfection and drug selection of genetically transformed blood stages of the rodent malaria parasite Plasmodium berghei. Nat Protoc 1: 346-356.

10. Desjardins RE, Canfield CJ, Haynes JD, Chulay JD (1979) Quantitative assessment of antimalarial activity in vitro by a semiautomated microdilution technique. Antimicrob Agents Chemother 16: 710-718.
